# Solving Problems on Graphs of High Rank-Width

**DOI:** 10.1007/s00453-017-0290-8

**Published:** 2017-02-13

**Authors:** Eduard Eiben, Robert Ganian, Stefan Szeider

**Affiliations:** Algorithms and Complexity Group, TU Wien, Vienna, Austria

**Keywords:** Fixed-parameter algorithms, Rank-width, Monadic second-order logic, Parameterized complexity

## Abstract

A modulator in a graph is a vertex set whose deletion places the considered graph into some specified graph class. The cardinality of a modulator to various graph classes has long been used as a structural parameter which can be exploited to obtain fixed-parameter algorithms for a range of hard problems. Here we investigate what happens when a graph contains a modulator which is large but “well-structured” (in the sense of having bounded rank-width). Can such modulators still be exploited to obtain efficient algorithms? And is it even possible to find such modulators efficiently? We first show that the parameters derived from such well-structured modulators are more powerful for fixed-parameter algorithms than the cardinality of modulators and rank-width itself. Then, we develop a fixed-parameter algorithm for finding such well-structured modulators to every graph class which can be characterized by a finite set of forbidden induced subgraphs. We proceed by showing how well-structured modulators can be used to obtain efficient parameterized algorithms for Minimum Vertex Cover and Maximum Clique. Finally, we use the concept of well-structured modulators to develop an algorithmic meta-theorem for deciding problems expressible in monadic second order logic, and prove that this result is tight in the sense that it cannot be generalized to LinEMSO problems.

## Introduction

Many important graph problems are known to be NP-hard, and yet admit efficient solutions in practice due to the inherent structure of instances. The parameterized complexity [16, 34] paradigm allows a more refined analysis of the complexity of various problems and hence enables the design of more efficient algorithms. In particular, given an instance of size *n* and a numerical parameter *k* which captures some property of the instance, one asks whether the instance can be solved in time $f(k)\cdot n^{\mathcal {O}(1)}$. Parameterized problems which admit such an algorithm are called *fixed-parameter tractable* (FPT), and the algorithms themselves are often called *fixed-parameter algorithms*.

Given the above, it is natural to ask what kind of structure can be exploited to obtain fixed-parameter algorithms for a wide range of natural graph problems. There are two very successful, mutually incomparable approaches which tackle this question.

**A:** **Width measures** Treewidth has become an extremely successful structural parameter with a wide range of applications in many fields of computer science. However, treewidth is not suitable for use in dense graphs. This led to the development of algorithms that use the parameter clique-width [11], which can be viewed as a relaxation of treewidth towards dense graphs. However, while there are efficient theoretical algorithms for computing tree decompositions, this is not the case for decompositions for clique-width. This shortcoming has later been overcome by the notion of rank-width [35], which improves upon clique-width by allowing the efficient computation of rank-decompositions while retaining all of the positive algorithmic results previously obtained for clique-width.
**B:** **Modulators** A modulator is a vertex set whose deletion places the considered graph into some specified graph class. A substantial amount of research has been placed into finding as well as exploiting small modulators to various graph classes [3, 18]. Popular notions such as vertex cover and feedback vertex set are also special cases of modulators (to the classes of edgeless graphs and forests, respectively). One advantage of parameterizing by the size of modulators is that it allows us to build on the vast array of research of polynomial-time algorithms on specific graph classes (see, for instance, [10, 33]). In other fields of computer science, modulators are often called *backdoors* and have been successfully used to obtain efficient algorithms for, e.g., satisfiability and constraint satisfaction [21, 22].

Our primary goal in this paper is to push the boundaries of tractability for a wide range of problems beyond the state of the art for both of these approaches. We summarize our contributions below.

1. We introduce a family of “hybrid” parameters that combine approaches A and B.

Given a graph *G* and a fixed graph class $\mathcal {H}$, the new parameters capture (roughly speaking) the minimum rank-width of any modulator of *G* into $\mathcal {H}$. We call this the *well-structure number* of *G* or $wsn^\mathcal {H}(G)$. The formal definition of the parameter also relies on the notion of *split decompositions* [13] to restrict the edges between the modulator and the rest of the graph; it is provided in Sect. 3, where we also prove that for any graph class $\mathcal {H}$ of unbounded rank-width, $wsn^\mathcal {H}$ is not larger and in many cases much smaller than both rank-width and the size of a modulator to $\mathcal {H}$.

2. We develop a fixed-parameter algorithm for computing $wsn^\mathcal {H}$.

As with most structural parameters, virtually all algorithmic applications of the well-structure number rely on having access to an appropriate decomposition. In Sect. 4 we provide a fixed-parameter algorithm for computing the $wsn^\mathcal {H}$ along with the corresponding decomposition for any graph class $\mathcal {H}$ which can be characterized by a finite set of forbidden induced subgraphs (*obstructions*). This is achieved by building on the polynomial algorithm for computing split-decompositions [27] in combination with the fixed-parameter algorithm for computing rank-width [29].

3. We design fixed-parameter algorithms for Minimum Vertex Cover (MinVC) and Maximum Clique (MaxClq) parameterized by $wsn^\mathcal {H}$.

Specifically, in Sect. 5 we show that for any graph class $\mathcal {H}$ characterizable by a finite obstruction set and admitting a polynomial-time algorithm for MinVC or MaxClq, there is a fixed-parameter algorithm solving MinVC or MaxClq (respectively) when parameterized by $wsn^\mathcal {H}$. We also give an overview of possible choices of $\mathcal {H}$ for MinVC and MaxClq.

4. We develop a *meta-theorem* to obtain fixed-parameter algorithms for problems definable in monadic second order (MSO) logic [11] parameterized by $wsn^\mathcal {H}$.

The meta-theorem requires that the problem is FPT when parameterized by the cardinality of a modulator to $\mathcal {H}$. We prove that this condition is not only necessary but also tight, in the sense that the weaker condition of polynomial-time tractability on $\mathcal {H}$ used for MinVC and MaxClq is not sufficient for FPT-time MSO model checking. Formal statements and proofs can be found in Sect. 6.

5. We show that, in general, solving LinEMSO problems [11, 19] is not FPT when parameterized by $wsn^\mathcal {H}$.

In particular, in Sect. 7 we give a proof that these problems remain NP-hard even on graphs of fixed $wsn^\mathcal {H}$ under the same conditions as those used for MSO model checking. This is somewhat surprising, since the fixed-parameter tractability of MSO optimization problems usually follows from the methods used for MSO model checking. On the other hand, there are strictly more classes of bounded width for our parameter than for rank-width and hence one cannot expect that every problem which is FPT parameterized by rank-width would remain FPT when parameterized by the well-structure number.

## Preliminaries

The set of natural numbers (that is, positive integers) will be denoted by $\mathbb {N}$. For $i \in \mathbb {N}$ we write [*i*] to denote the set $\{1, \ldots , i \}$. If $\sim$ is an equivalence relation over a set *A*, then for $a\in A$ we use $[a]_{\sim }$ to denote the equivalence class containing *a*.

### Graphs

We will use standard graph theoretic terminology and notation (cf. [15]). All graphs we consider are finite, simple and undirected. The non-leaf vertices of a tree are called its *internal nodes*. If *S* is a set of leaves of a tree *T*, then *T*(*S*) denotes the smallest connected subtree spanning *S*.

Given a graph $G=(V(G),E(G))$ and $A\subseteq V(G)$, we denote by *N*(*A*) the set of neighbors of *A* in $V(G){\setminus } A$; if *A* contains a single vertex *v*, we use *N*(*v*) instead of $N(\{v\})$. We use *V* and *E* as shorthand for *V*(*G*) and *E*(*G*), respectively, when the graph is clear from context. Two vertex sets *A*, *B* are *overlapping* if $A\cap B, A{\setminus } B$, and $B{\setminus } A$ are all nonempty. $G-A$ denotes the subgraph of *G* obtained by deleting *A*.

Given a graph $G=(V,E)$ and a graph class $\mathcal {H}$, a set $X\subseteq V$ is called a *modulator* to $\mathcal {H}$ if $G-X\in \mathcal {H}$. A graph class is called *hereditary* if it is closed under vertex deletion. A graph *H* is an *induced subgraph* of *G* if *H* can be obtained by deleting vertices (along with all of their incident edges) from *G*. For $A\subseteq V(G)$ we use *G*[*A*] to denote the subgraph of *G* obtained by deleting $V(G){\setminus } A$. Let $\mathcal {F}$ be a finite set of graphs; then the class of $\mathcal {F}$-*free* graphs is the class of all graphs which do not contain any graph in $\mathcal {F}$ as an induced subgraph. We will often refer to elements of $\mathcal {F}$ as *obstructions*, and we say that the class of $\mathcal {F}$-free graphs is *characterized by* $\mathcal {F}$.

### Fixed-Parameter Tractability

We refer the reader to the standard textbooks [14, 16, 34] for an introduction to parameterized complexity. A *parameterized problem* $\mathcal {P}$ is a subset of $\varSigma ^* \times \mathbb {N}$ for some finite alphabet $\varSigma$. For a problem instance $(x,k) \in \varSigma ^* \times \mathbb {N}$ we call *x* the main part and *k* the parameter.

A parameterized problem $\mathcal {P}$ is *fixed-parameter tractable* (FPT in short) if a given instance (*x*, *k*) can be solved in time $f(k) \cdot |x|^{\mathcal {O}(1)}$ where *f* is an arbitrary computable function of *k*. Algorithms with running time in this form are called *fixed-parameter algorithms*, and we also slightly abuse notaton and refer to this runtime simply as *FPT time*.

A parameterized problem $\mathcal {P}$ is *paraNP-hard* if the unparameterized problem corresponding to the restriction of $\mathcal {P}$ to some constant value of the parameter is NP-hard. For instance, the classical Minimum Vertex Cover problem parameterized by the degree of a graph is paraNP-hard, since Minimum Vertex Cover remains NP-hard even on graphs of degree at most 3.

### Splits and Graph Labeled Trees

The notions and terminology introduced in this subsection play an important role in particular when formally defining our parameters (Sect. 3) and for computing them (Sect. 3). The contents of this subsection are based on the original work of Cunningham [13] as well as on more recent results by several authors [25–27].

A *split* of a connected graph $G=(V,E)$ is a vertex bipartition $\{A,B\}$ of *V* such that every vertex of $A' = N(B)$ has the same neighborhood in $B'=N(A)$. The sets $A'$ and $B'$ are called *frontiers* of the split.

A split is said to be *non-trivial* if both sides have at least two vertices. A connected graph which does not contain a non-trivial split is called *prime*. A bipartition is *trivial* if one of its parts is the empty set or a singleton. Cliques and stars are called *degenerate* graphs; notice that every non-trivial bipartition of their vertices is a split.

Let $G=(V,E)$ be a graph. To simplify our exposition, we will use the notion of *split-modules* instead of splits where suitable. A set $A\subseteq V$ is called a *split-module* of *G* if there exists a connected component $G'=(V',E')$ of *G* such that $A\subseteq V'$ and $\{A,V'{\setminus } A\}$ forms a split of $G'$. Notice that if *A* is a split-module then *A* can be partitioned into $A_1$ and $A_2$ such that $N(A_2)\subseteq A$ and for each $v_1, v_2\in A_1$ it holds that $N(v_1)\cap (V'{\setminus } A)=N(v_2)\cap (V'{\setminus } A)$. For technical reasons, *V* and $\emptyset$ are also considered split-modules. We say that two disjoint split-modules $X, Y \subseteq V$ are *adjacent* if there exist $x \in X$ and $y \in Y$ such that *x* and *y* are adjacent.

Our algorithms for computing well-structured modulators will rely on two deep results related to splits, specifically Theorems 1 and 2. To formally state these theorems, we will need to introduce a bit of extra notation.

A *graph-labeled tree* (which can be viewed as a more modern approach to the previously used *split-decompositions*) is a pair $(T, \mathcal {F})$, where *T* is a tree and $\mathcal {F}$ is a set of graphs such that each internal node *u* of *T* is *labeled* by a graph $G(u)\in \mathcal {F}$ and there is a bijection between the edges of *T* incident to *u* and vertices of *G*(*u*). When clear from the context, we may use *u* as a shorthand for $G(u) \in \mathcal {F}$; for instance, we use *V*(*u*) to denote *V*(*G*(*u*)) and we say that an edge of *T* incident to *u* is *incident* to the vertex of *G*(*u*) mapped to it. Graph-labeled trees were introduced by Gioan and Paul [25, 26], and in the following paragraphs we recall some useful definitions and facts that also appeared in follow-up work [27].

For an internal node *u* of *T*, the vertices of *V*(*u*) are called *marker* vertices and the edges of *E*(*u*) are called *label-edges*. Edges of *T* incident to two internal nodes are called *tree-edges*. Marker vertices incident to a tree-edge *e* are called the *extremities* of *e*, and each leaf *v* is *associated with* the unique marker vertex *q* (in the neighbor of *v*) mapped to the edge incident to *v*. Perhaps the most important notion for graph-labeled trees with respect to split decomposition is that of *accessibility*.

#### Definition 1

Let $(T, \mathcal {F})$ be a graph-labeled tree. The marker vertices *q* and $q'$ are accessible from one another if there is a sequence $\varPi$ of marker vertices $q,\ldots ,q'$ such that the two following conditions holds.

1. Every two consecutive elements of $\varPi$ are either the vertices of a label-edge or the extremities of a tree-edge;
2. the sequence of edges obtained above alternates between tree-edges and label-edges.

**Fig. 1:**
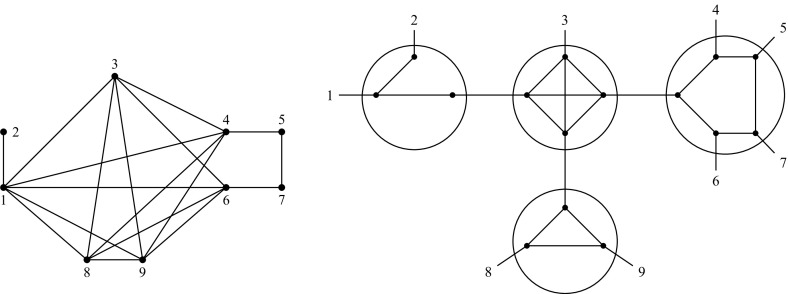
A graph-labeled tree (*right*) and its accessibility graph (*left*)

Two leaves are accessible if their associated marker vertices are accessible. The *accessibility graph* of a graph-labeled tree $(T,\mathcal {F})$, denoted $Gr(T,\mathcal {F})$, is the graph whose vertices are leaves of *T* and which has an edge between two distinct leaves *l* and $l'$ if and only if they are accessible from one another. Conversely, we may say that $(T,\mathcal {F})$ is the graph-labeled tree of $Gr(T,\mathcal {F})$. See Fig. 1 for an example.

#### Definition 2

([27]) Let *e* be a tree-edge incident to internal nodes *u* and $u'$ in a graph-labeled tree, and let $q\in V (u)$ and $q' \in V (u')$ be the extremities of *e*. The *node-join* of $u, u'$ replaces *u* and $u'$ with a new internal node *v* labeled by the graph formed from the disjoint union of *G*(*u*) and $G(u')$ as follows: all possible label-edges are added between *N*(*q*) and $N (q' )$, and then *q* and $q'$ are deleted. The new node *v* is made adjacent to all neighbors of *u* and $u'$ in *T*. The *node-split* is then the inverse of the node-join.

Notice that the node-join operation and the node-split operation preserve the accessibility graph of the graph-labeled tree. A graph-labeled tree is reduced if all its labels are either prime or degenerate, and no node-join of two cliques or two stars $S_1$ and $S_2$ where the center of $S_1$ is adjacent to a leaf of $S_2$ is not possible. It is known that for every connected graph *G*, there exists a unique reduced graph-labeled tree $(T , \mathcal {F})$ such that $G = Gr(T , \mathcal {F})$ [13, 25–27]; this unique reduced graph-labeled tree is called the *split-tree* and is denoted $\text {ST}(G)$.

#### Theorem 1

([4, 13, 25–27]) Let $(T , \mathcal {F})$ be the split-tree of a connected graph *G*. Every split of *G* is the bipartition (of leaves) induced by removing an internal tree-edge from $T'$, where $T' = T$ or $T'$ is obtained from *T* by exactly one node-split of a degenerate node.

#### Theorem 2

([9, 27]) The split-tree $\text {ST}(G)$ of a connected graph *G* having *n* vertices and *m* edges can be built incrementally in time $\mathcal {O}((n +m)\alpha (n + m))$, where $\alpha$ is the inverse Ackermann function.

### Rank-Width

Rank-width was introduced by Oum and Seymour [35] and is closely related to clique-width. To define it, we first need to introduce the *bipartite adjacency matrix* $\varvec{A}_G[U,W]$.

For a graph *G* and $U,W\subseteq V(G)$, let $\varvec{A}_G[U,W]$ denote the $U\times W$-submatrix of the adjacency matrix over the two-element field $\mathrm {GF}(2)$, i.e., the entry $a_{u,w}, u\in U$ and $w\in W$, of $\varvec{A}_G[U,W]$ is 1 if and only if $\{u,w\}$ is an edge of *G*. The *cut-rank* function $\rho _G$ of a graph *G* is defined as follows: For a bipartition (*U*, *W*) of the vertex set $V(G), \rho _G(U)=\rho _G(W)$ equals the rank of $\varvec{A}_G[U,W]$ over $\mathrm {GF}(2)$. We note that $\rho _G$ is a symmetric function, and observe that a split-module *X* can be seen as a subgraph such that $\varvec{A}_G[X,V(G){\setminus } X]=1$.

A *rank-decomposition* of a graph *G* is a pair $(T,\mu )$ where *T* is a tree of maximum degree 3 and $\mu :V(G)\rightarrow \{t: \hbox {} t \hbox {is a leaf of} T\}$ is a bijective function. For an edge *e* of *T*, the connected components of $T - e$ induce a bipartition (*X*, *Y*) of the set of leaves of *T*. The *width* of an edge *e* of a rank-decomposition $(T,\mu )$ is $\rho _G(\mu ^{-1} (X))$. The *width* of $(T,\mu )$ is the maximum width over all edges of *T*. The *rank-width* of *G*, *rw*(*G*) in short, is the minimum width over all rank-decompositions of *G*. A graph class $\mathcal {H}$ is of *unbounded rank-width* if for each $i\in \mathbb {N}$ there exists a graph $G\in \mathcal {H}$ such that $rw(G)> i$.

**Fig. 2:**
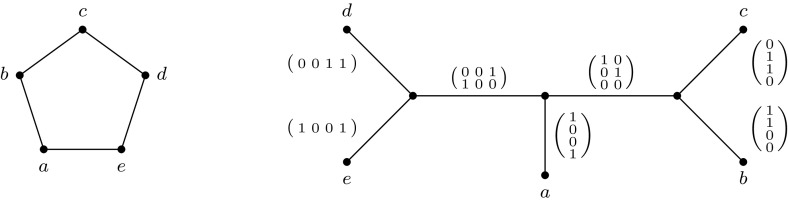
A rank-decomposition of the cycle $C_5$

An example of a rank-decomposition is provided in Fig. 2.

#### Theorem 3

([29]) Let $k \in \mathbb {N}$ and $n \ge 2$. For an *n*-vertex graph *G*, we can output a rank-decomposition of width at most *k* or confirm that the rank-width of *G* is larger than *k* in time $f(k)\cdot n^3$, where *f* is a computable function.

### Monadic Second Order Logic on Graphs

Here we introduce monadic second order logic, which will play a crucial role in our positive (Sect. 6) as well as negative (Sect. 7) algorithmic results.

We assume that we have an infinite supply of individual variables, denoted by lowercase letters *x*, *y*, *z*, and an infinite supply of set variables, denoted by uppercase letters *X*, *Y*, *Z*. *Formulas* of *monadic second-order logic* (MSO) are constructed from atomic formulas *E*(*x*, *y*), *X*(*x*), and $x = y$ using the connectives $\lnot$ (negation), $\wedge$ (conjunction) and existential quantification $\exists x$ over individual variables as well as existential quantification $\exists X$ over set variables. Individual variables range over vertices, and set variables range over sets of vertices. The atomic formula *E*(*x*, *y*) expresses adjacency, $x = y$ expresses equality, and *X*(*x*) expresses that the vertex *x* is in the set *X*. From this, we define the semantics of monadic second-order logic in the standard way (this logic is sometimes called ${\text {MSO}} _1$).

*Free and bound variables* of a formula are defined in the usual way. A *sentence* is a formula without free variables. We write $\varphi (X_1, \ldots , X_n)$ to indicate that the set of free variables of formula $\varphi$ is $\{X_1, \ldots , X_n\}$. If $G = (V,E)$ is a graph and $S_1, \ldots , S_n \subseteq V$ we write $G \models \varphi (S_1, \ldots , S_n)$ to denote that $\varphi$ holds in *G* if the variables $X_i$ are interpreted by the sets $S_i$, for $i \in [n]$. For a fixed MSO sentence $\varphi$, the MSO model checking problem ($\textsc {MSO-MC}_{\varphi }$) asks whether an input graph *G* satisfies $G\models \varphi$.

It is known that MSO formulas can be checked in uniformly polynomial time on graphs of bounded rank-width.

#### Theorem 4

([19]) Let $\varphi$ and $\psi =\psi (X)$ be fixed MSO formulas. There exists a computable function *f* and an algorithm such that, given an *n*-vertex graph *G* and $S\subseteq V(G)$, decides whether $G \models \varphi$ and whether $G\models \psi (S)$ in time $f(rw(G))\cdot n^3$.

We review MSO-*types* roughly following the presentation in the textbook by Libkin [32]. The *quantifier rank* of an MSO formula $\varphi$ is defined as the nesting depth of quantifiers in $\varphi$. For non-negative integers *q* and *l*, let ${\text {MSO}} _{q,l}$ consist of all MSO formulas of quantifier rank at most *q* having at most *l* free set variables.

Let $\varphi = \varphi (X_1,\ldots ,X_l)$ and $\psi = \psi (X_1,\ldots ,X_l)$ be MSO formulas. We say $\varphi$ and $\psi$ are *equivalent*, written $\varphi \equiv \psi$, if for all graphs *G* and $U_1, \ldots , U_l \subseteq V(G), G \models \varphi (U_1,\ldots , U_l)$ if and only if $G \models \psi (U_1,\ldots , U_l)$. Given a set *F* of formulas, let ${F/\mathord \equiv }$ denote the set of equivalence classes of *F* with respect to $\equiv$. A *system of representatives* of $F/\mathord \equiv$ is a set $R \subseteq F$ such that $R \cap C \ne \emptyset$ for each equivalence class $C \in F/\mathord \equiv$. The following statement has a straightforward proof using normal forms (see [32, Proposition 7.5 and Lemma 3.13] for details).

#### Fact 1

Let *q* and *l* be fixed non-negative integers. The set ${\text {MSO}} _{q,l}/\mathord \equiv$ is finite, and one can compute a finite system of representatives of ${\text {MSO}} _{q,l}/\mathord \equiv$.

Note that the system of representatives obtained in this way need not be inclusion-minimal, and we do not assume to have a mapping from this system of representatives to elements of ${{\text {MSO}} _{q,l}/\mathord \equiv }$. We will assume that for every pair of non-negative integers *q* and *l* the system of representatives of ${\text {MSO}} _{q,l}/\mathord \equiv$ given by Fact 1 is fixed.

#### Definition 3

*(MSO Type)* Let *q*, *l* be non-negative integers. For a graph *G* and an *l*-tuple $\mathbf {U}$ of sets of vertices of *G*, we define MSO-$type _q(G,\mathbf {U})$ as the set of formulas $\varphi \in {\text {MSO}} _{q,l}$ such that $G \models \varphi (\mathbf {U})$. We call MSO-$type _q(G,\mathbf {U})$ the MSO *q*-*type of* $\mathbf {U}$ *in* *G*.

Since we will only be dealing with MSO logic, throughout the paper we will refer to MSO-types simply as *types*. It follows from Fact 1 that up to logical equivalence, every type contains only finitely many formulas. This allows us to represent types using MSO formulas, as formalized in the next lemma. We remark that the statement of the next lemma used in previous work [20] did not specify the (“fixed-parameter”) dependence of the running time on the rank-width, and so here we give a proof of the lemma for completeness.

#### Lemma 1

([20]) Let *q* and *l* be non-negative integer constants. Let *G* be a graph, and let $\mathbf {U}$ be an *l*-tuple of sets of vertices of *G*. One can compute a formula $\varPhi \in {\text {MSO}} _{q,l}$ such that for any graph $G'$ and any *l*-tuple $\mathbf {U}'$ of sets of vertices of $G'$ we have $G' \models \varPhi (\mathbf {U}')$ if and only if $type _q(G,\mathbf {U}) = type _q(G',\mathbf {U}')$. Moreover, $\varPhi$ can be computed in time $f(rw(G))\cdot |V|^{3}$.

#### Proof

Let *R* be a system of representatives of ${\text {MSO}} _{q,l}/\mathord \equiv$ given by Fact 1. Because *q* and *l* are constant, we can consider both the cardinality of *R* and the time required to compute it as constants. Let $\varPhi \in {\text {MSO}} _{q,l}$ be the formula defined as $\varPhi = \bigwedge _{\varphi \in S} \varphi \wedge \bigwedge _{\varphi \in R {\setminus } S} \lnot \varphi$, where $S = \{\,\varphi \in R \;{|}\;G \models \varphi (\mathbf {U}) \,\}$. We can compute $\varPhi$ by deciding $G \models \varphi (\mathbf {U})$ for each $\varphi \in R$. Since the number of formulas in *R* is a constant, this can be done in time $f(rw(G))\cdot |V|^{3}$ (for some computable function *f*) as checking whether *G* satisfied $\varphi (\mathbf {U})$ can be done in time $q(rw(G))\cdot |V|^{3}$ (for some computable function *q*).

Let $G'$ be an arbitrary graph and let $\mathbf {U}'$ be an *l*-tuple of subsets of $V(G')$. We claim that $type _q(G, \mathbf {U}) = type _q(G', \mathbf {U'})$ if and only if $G' \models \varPhi (\mathbf {U}')$. Since $\varPhi \in {\text {MSO}} _{q,l}$ the forward direction is trivial. For the converse, assume $type _q(G, \mathbf {U}) \ne type _q(G', \mathbf {U'})$. First suppose $\varphi \in type _q(G, \mathbf {U}) {\setminus } type _q(G', \mathbf {U'})$. The set *R* is a system of representatives of ${\text {MSO}} _{q,l}/\mathord \equiv$ , so there has to be a $\psi \in R$ such that $\psi \equiv \varphi$. But $G' \models \varPhi (\mathbf {U}')$ implies $G' \models \psi (\mathbf {U}')$ by construction of $\varPhi$ and thus $G' \models \varphi (\mathbf {U}')$, a contradiction. Now suppose $\varphi \in type _q(G', \mathbf {U}') {\setminus } type _q(G, \mathbf {U})$. An analogous argument proves that there has to be a $\psi \in R$ such that $\psi \equiv \varphi$ and $G' \models \lnot \psi (\mathbf {U}')$. It follows that $G' \not \models \varphi (\mathbf {U}')$, which again yields a contradiction. $\square$

The remainder of the section introduces the classical notion of MSO games (Definition 5) and their relation to MSO types (Theorem 5). However, to formally define MSO games, we first need the notion of partial isomorphism.

#### Definition 4

*(Partial isomorphism)* Let $G, G'$ be graphs, and let $\mathbf {V} = (V_1, \ldots , V_l)$ and $\mathbf {U} = (U_1, \ldots , U_l)$ be tuples of sets of vertices such that $V_i \subseteq V(G)$ and $U_i \subseteq V(G')$ for each $i \in [l]$. Let $\mathbf {v} = (v_1, \ldots , v_m)$ and $\mathbf {u} = (u_1, \ldots , u_m)$ be tuples of vertices such that $v_i \in V(G)$ and $u_i \in V(G')$ for each $i \in [m]$. Then $(\mathbf {v}, \mathbf {u})$ defines a *partial isomorphism between* $(G, \mathbf {V})$ *and* $(G', \mathbf {U})$ if the following two conditions hold:

- For every $$i,j \in [m]$$,
  $$\begin{aligned} v_i = v_j \, \Leftrightarrow \, u_i = u_j \text { and } v_iv_j \in E(G)\, \Leftrightarrow \, u_iu_j \in E(G'). \end{aligned}$$
- For every $i \in [m]$ and $j \in [l]$,
  $$\begin{aligned} v_i \in V_j \, \Leftrightarrow u_i \in U_j. \end{aligned}$$

In the definition of MSO games given below, we denote the concatenation of tuple $\mathbf {A}$ by tuple $\mathbf {B}$ as $\mathbf {A}\frown \mathbf {B}$.

#### Definition 5

([32], Definition 7.6) Let *G* and $G'$ be graphs, and let $\mathbf {V_0}$ be a *k*-tuple of subsets of *V*(*G*) and let $\mathbf {U_0}$ be a *k*-tuple of subsets of $V(G')$. Let *q* be a non-negative integer. The *q*-*round* MSO *game on* *G* *and* $G'$ *starting from* $(\mathbf {V_0}, \mathbf {U_0})$ is played as follows. The game proceeds in rounds, and each round consists of one of the following kinds of moves.

- **Point move:** The Spoiler picks a vertex in either *G* or $G'$; the Duplicator responds by picking a vertex in the other graph.
- **Set move:** The Spoiler picks a subset of *V*(*G*) or a subset of $V(G')$; the Duplicator responds by picking a subset of the vertex set of the other graph.

Let $\mathbf {v}=(v_1,\ldots ,v_m), v_i \in V(G)$ and $\mathbf {u}=(u_1,\ldots ,u_m), u_i \in V(G')$ be the point moves played in the *q*-round game, and let $\mathbf {V}=(V_1, \ldots , V_l), V_i\subseteq V(G)$ and $\mathbf {U}=(U_1,\ldots , U_l), U_i \subseteq V(G')$ be the set moves played in the *q*-round game, so that $l + m = q$ and moves belonging to same round have the same index. Then the Duplicator wins the game if $(\mathbf {v}, \mathbf {u})$ is a partial isomorphism of $(G, \mathbf {V_0}\frown \mathbf {V})$ and $(G', \mathbf {U_0}\frown \mathbf {U})$. If the Duplicator has a winning strategy, we write $(G, \mathbf {V_0}) \equiv ^{{\text {MSO}}}_q (G', \mathbf {U_0})$.

#### Theorem 5

([32], Theorem 7.7) Given two graphs *G* and $G'$ and two *l*-tuples $\mathbf {V_0}, \mathbf {U_0}$ of sets of vertices of *G* and $G'$, respectively, we have

$$\begin{aligned} type _q(G, \mathbf {V_0}) = type _q(G', \mathbf {U_0}) \, \Leftrightarrow \, (G, \mathbf {V_0}) \equiv ^{{\text {MSO}}}_q (G', \mathbf {U_0}). \end{aligned}$$

## Well-Structured Modulators

Recall that a modulator to a graph class $\mathcal {H}$ is a vertex-subset of a graph *G* such that its deletion puts *G* into $\mathcal {H}$ (see Sect. 2.1).

### Definition 6

Let $\mathcal {H}$ be a hereditary graph class and let *G* be a graph. A set $\mathbf {X}$ of pairwise-disjoint split-modules of *G* is called a *k*-*well-structured modulator* to $\mathcal {H}$ if

1. $$|\mathbf {X}|\le k$$, and
2. $\bigcup _{X_i\in \mathbf {X}} X_i$ is a modulator to $\mathcal {H}$, and
3. $$rw(G[X_i])\le k$$ for each $$X_i\in \mathbf {X}$$.

**Fig. 3:**
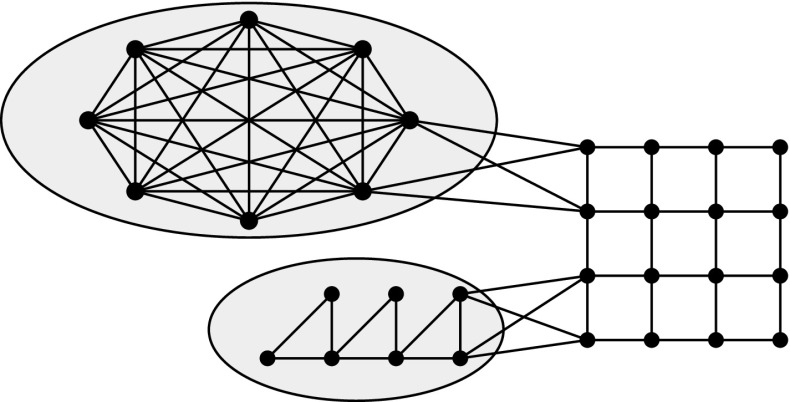
A graph with a 2-well-structured modulator to $K_3$-free graphs (*in the two shaded areas*)

An example of a 2-well-structured modulator is provided in Figure 3. For the sake of brevity and when it is clear from the context, we will sometimes identify $\mathbf {X}$ with $\bigcup _{X_i\in \mathbf {X}} X_i$ (for instance $G-\mathbf {X}$ is shorthand for $G-\bigcup _{X_i\in \mathbf {X}} X_i$). To allow a concise description of our parameters, for any hereditary graph class $\mathcal {H}$ we let the *well-structure number* ($wsn^{\mathcal {H}}$ in short) denote the minimum *k* such that *G* has a *k*-well-structured modulator to $\mathcal {H}$. Similarly, we let $mod^{\mathcal {H}}(G)$ denote the minimum *k* such that *G* has a modulator of cardinality *k* to $\mathcal {H}$.

### Proposition 1

Let $\mathcal {H}$ be an arbitrary hereditary graph class of unbounded rank-width.

1. $rw(G)\ge wsn^\mathcal {H}(G)$ for every graph *G*. Furthermore, for every $i\in \mathbb {N}$ there exists a graph $G_i$ such that $rw(G_i)\ge wsn^\mathcal {H}(G_i)+i$, and
2. $mod^\mathcal {H}(G)\ge wsn^\mathcal {H}(G)$ for every graph *G*. Furthermore, for every $i\in \mathbb {N}$ there exists a graph $G_i$ such that $mod^\mathcal {H}(G_i)\ge wsn^\mathcal {H}(G_i)+i$.

### Proof

1. For $rw(G)\ge wsn^\mathcal {H}(G)$ notice that for every graph *G* of rank-width *k*, the set $\{V(G)\}$ is a *k*-well-structured modulator to the empty graph. For the second claim, since $\mathcal {H}$ has unbounded rank-width, for every $i\in \mathbb {N}$ it contains some graph $G_i$ such that $rw(G_i)>i$; by definition, $wsn^\mathcal {H}(G_i)=0$.
2. For $mod^\mathcal {H}(G)\ge wsn^\mathcal {H}(G)$, let *G* be a graph containing a modulator $X=\{v_1,\ldots ,$ $v_k\}$ to $\mathcal {H}$. It is easy to check that $\mathbf {X}=\{\{v_1\},\ldots ,\{v_k\}\}$ is a *k*-well-structured modulator to $\mathcal {H}$. For the second claim, let $G'\not \in \mathcal {H}$ and let $k=rw(G')$. Consider the graph $G_i$ consisting of $i+1+k$ disjoint copies of $G'$ and a vertex *q* which is adjacent to every other vertex of *G*. Since $\mathcal {H}$ is hereditary, we may assume without loss of generality that it contains the single-vertex graph. It is then easy to check that $\{V(G){\setminus } \{q\}\}$ forms a *k*-well-structured modulator in *G* to $\mathcal {H}$. Now consider an arbitrary set $X\subseteq V(G)$ of cardinality at most $i+k$. Clearly, there must exist some copy of $G'$, say $G'_j$, such that $X\cap V(G'_j)=\emptyset$. Since $G'_j\not \in \mathcal {H}$, it follows from the hereditarity of $\mathcal {H}$ that $G-X\not \in \mathcal {H}$ and hence *X* cannot be a modulator to $\mathcal {H}$. We conclude $mod^\mathcal {H}(G_i)>i+k=i+wsn^\mathcal {H}(G_i)$.

$$\square $$

## Finding Well-Structured Modulators

The objective of this subsection is to prove the following theorem.

### Theorem 6

Let $\mathcal {H}$ be a graph class characterized by a finite obstruction set. There exists a fixed-parameter algorithm parameterized by *k* which for every input graph *G* either finds a *k*-well-structured modulator to $\mathcal {H}$, or detects that no such *k*-well-structured modulator exists.

Interestingly, the techniques we will use to prove Theorem 6 only work if the rank-width of the graph is sufficiently large. This is not a problem though, since on graphs of small rank-width we can always directly use rank-width to find *k*-well-structured modulators.

Our first course of action is the statement of several useful properties of splits in graphs. We remark that for most of this section we will restrict ourselves to connected graphs, and show how to deal with general graphs later on; this allows us to use the following result by Cunningham.

### Theorem 7

([13]) Let $\{ A, C\}, \{ B, D\}$ be splits of a connected graph *G* such that $|A\cap B|\ge 2$ and $A \cup B\ne V(G)$. Then $\{A\cap B, C\cup D\}$ is a split of *G*.

### Lemma 2

If *A* and *B* are overlapping split-moduleof a connected graph $G=(V,E)$, then $A\cup B$ is also a split-module. Moreover, if $A\cup B\ne V$, then also $A\cap B$ is a split-module.

### Proof

If $V=A\cup B$, then $A\cup B$ is clearly a split-module. So, assume $A\cup B\ne V$ and let $C=V{\setminus } A$ and $D=V{\setminus } B$; note that $C\cup D\ne V$ since *A*, *B* are overlapping. We make the following exhaustive case distinction:

- if $|A\cap B|=1$ and $|C\cap D|=1$, then both $A\cap B$ and $A\cup B=V{\setminus } (C\cap D)$ are easily seen to be split-modules;
- if $|A\cap B|\ge 2$ and $|C\cap D|=1$, then $A\cap B$ is a split-module by Theorem 7 and $A\cup B$ is also a split-module because $C\cap D$ is a split-module;
- if $|A\cap B|=1$ and $|C\cap D|\ge 2$, then $A\cap B$ is a split-module and $A\cup B$ is also a split-module because *C*, *D* satisfy the conditions of Theorem 7 and hence $C\cap D = V{\setminus } (A\cup B)$ forms a split-module;
- if $|A\cap B|\ge 2$ and $|C\cap D|\ge 2$, then $A\cap B$ is a split-module by Theorem 7 and $A\cup B$ is also a split-module because *C*, *D* satisfy the conditions of Theorem 7, as in the previous case.

$$\square $$

### Lemma 3

Let $G=(V,E)$ be a connected graph and *A*, *B* be overlapping split-modules. Then $A{\setminus } B$ is also a split-module.

### Proof

The lemma clearly holds if $|A{\setminus } B|\le 1$, so we may assume that $|A{\setminus } B|\ge 2$. Let $Z=V{\setminus } B$; since *B* is a split module, so is *Z*. Furthermore, since *A* and *B* are overlapping, it holds that $B{\setminus } A$ is nonempty and hence $V\ne Z\cup A$. Since $Z\cap A=A{\setminus } B$, we have $|Z\cap A|\ge 2$ and hence we conclude that $Z\cap A=A{\setminus } B$ is a split module by Theorem 7. $\square$

### Lemma 4

Let $k\in \mathbb {N}, G=(V,E)$ be a graph, and *A*, *B*, *C* be pairwise disjoint split-modules such that $A\cup B\cup C=V$. Let *a*, *b*, *c* be arbitrary vertices such that $a\in N(A), b\in N(B)$, and $c\in N(C)$. If $\max \big (rw(G[A\cup \{a\}]),rw(G[B\cup \{b\}]),rw(G[C\cup \{c\}])\big )\le k$, then $rw(G)\le k$.

### Proof

Let $\mathcal {T}_{A} = (T_{A}, \mu _{A}), \mathcal {T}_{B} = (T_{B}, \mu _{B})$, and $\mathcal {T}_{C} = (T_{C}, \mu _{C})$ be witnessing rank decompositions of *G*[*A*], *G*[*B*], and *G*[*C*], respectively.

We construct a rank decomposition $\mathcal {T} = (T, \mu )$ of *G* as follows.

Let $l_{a}$ be the leaf (note that $\mu _{A}$ is bijective) of $T_{A}$ such that $\mu _{A}(a) = l_{a}$. Similarly, let $l_b$ and $l_c$ be the leaves such that $\mu _B(b)=l_b$ and $\mu _C(c)=l_c$, respectively. We obtain *T* from $T_{A}$ by adding disjoint copies of $T_{B}$ and $T_{C}$ and then identifying $l_{a}$ with the copies of $l_{b}$ and $l_{b}$. Since $T_{A}, T_{B}$, and $T_{C}$ are subcubic, so is *T*.

We define the mapping $\mu : V(G) \rightarrow \{\,t \;{|}\;$ t is a leaf of $T \,\}$ by

$$\begin{aligned} \mu (v) = {\left\{ \begin{array}{ll} \mu _{a}(v) &{}\text {if }v \in A, \\ c(\mu _{b}(v)) &{}\text {if }v \in B, \\ c(\mu _{c}(v)) &{}\text {otherwise,} \end{array}\right. } \end{aligned}$$

where *c* maps internal nodes in $T_{B} \cup T_{C}$ to their copies in *T*. The mappings $\mu _{A}, \mu _{B}$, and $\mu _{C}$ are bijections and *c* is injective, so $\mu$ is injective. By construction, the image of *V*(*G*) under $\mu$ is the set of leaves of *T*, so $\mu$ is a bijection. Thus $\mathcal {T} = (T, \mu )$ is a rank decomposition of *G*.

We prove that the width of $\mathcal {T}$ is at most *k*. Given a rank decomposition $\mathcal {T}^* = (T^*, \mu ^*)$ and an edge *e* of $T^*$, the connected components of $T^* - e$ induce a bipartition (*X*, *Y*) of the leaves of $T^*$. We set $f: (\mathcal {T}^*, e) \mapsto ({\mu ^*}^{-1}(X), {\mu ^*}^{-1}(Y))$. Take any edge *e* of *T*. There is a natural bijection $\beta$ from the edges in *T* to the edges of $T_{A} \cup T_{B} \cup T_{C}$. Accordingly, we distinguish three cases for $e ' = \beta (e)$:

1. $e' \in T_{A}$. Let $(U, W) = f(\mathcal {T}_{A}, e')$. Without loss of generality assume that $a \in W$. Then by construction of $\mathcal {T}$ , we have $f(\mathcal {T}, e) = (U, W \cup B\cup C)$. Let $u\in A$ and $v\in B\cup C$. Since *A* is split-module either $v\notin N(A)$ and $\mathbf {A}_G(u, v) =0$ for all $u\in A$, or $v\in N(A)$ in which case $\mathbf {A}_G(u, v) = \mathbf {A}_G(u, a)$ for all $u\in A$. Therefore, to obtain $\mathbf {A}_G(U, W\cup B\cup C)$ one can simply copy the column corresponding to *a* in $\mathbf {A}_G(U, W)$ or add some empty columns. This does not increase the rank of the matrix.
2. $e' \in T_{B}$. This case is symmetric to case 1, with *A* and *B* switching their roles and *b* taking the role of *a*.
3. $e' \in T_{C}$. This case is symmetric to case 1, with *A* and *C* switching their roles and *c* taking the role of *a*.

Since $\beta$ is bijective, this proves that the rank of any bipartite adjacency matrix induced by removing an edge $e \in T$ is bounded by *k*. We conclude that the width of $\mathcal {T}$ is at most *k* and thus $rw(G) \le k$. $\square$

By repeating the proof technique of Lemma 4 without the set *C*, we obtain the following corollary.

### Corollary 1

Let $k\in \mathbb {N}, G=(V,E)$ be a graph, and *A*, *B* pairwise disjoint split-modules such that $A\cup B=V$. Let $a,b\in V$ be such that $a\in N(A)$ and $b\in N(B)$. If $\max \big (rw(G[A\cup \{a\}]),rw(G[B\cup \{b\}])\big )\le k$, then $rw(G)\le k$.

### Lemma 5

Let $k \in \mathbb {N}$ and let $G=(V,E)$ be a connected graph having split-modules $M_1, M_2$ where $M_1 \cup M_2 = V$ and $\max (rw(G[M_1]), rw(G[M_2])) \le k$. Then $rw(G) \le k+1$.

### Proof

Let $M_{22} = M_2{\setminus } M_1$. Clearly, $\{M_1, M_{22}\}$ is a split. Since rank-width is preserved by taking induced subgraphs, the graph $G[M_{22}]$ has rank-width at most *k*. Let $v_1\in N(M_{22})$ and $v_2\in N(M_1)$. It is easy to see that graphs $G_{1} = G[M_{1} \cup \{v_{2}\}]$ and $G_{2} = G[M_{22} \cup \{v_{1}\}]$ have rank-width at most $k+1$. We finish the proof by applying Corollary 1, using $M_1, M_{22}$ in roles of *A*, *B* and $v_1, v_2$ in roles of *a*, *b*, respectively. $\square$

The following lemma in essence shows that the relation of being in a split-module of small rank-width is transitive (assuming sufficiently high rank-width). The significance of this will become clear later on.

### Lemma 6

Let $k \in \mathbb {N}$. Let $G=(V,E)$ be a connected graph of rank-width at least $k+2$ and let $M_1, M_2$ be split-modules of *G* such that $M_1 \cap M_2 \ne \emptyset$ and $\max (rw(G[M_1]), rw(G[M_2])) \le k$. Then $M_1 \cup M_2$ is a split-module of *G* and $rw(G[M_1\cup M_2]) \le k$.

### Proof

If $M_1 \subseteq M_2$ or $M_2 \subseteq M_1$ the result is immediate, hence we may assume that they are overlapping. Lemma 5 and $rw(G)\ge k+2$ together imply that $M_1\cup M_2 \ne V$. Let $M_{11} = M_1 {\setminus } M_2, M_{22} = M_2 {\setminus } M_1$, and $M_{12} = M_1 \cap M_2$. It follows from Lemmas 2 and 3 that these sets are split-modules of *G*. Let $v_{11} \in N(V{\setminus } M_{11}), v_{22} \in N(V{\setminus } M_{22})$, and $v_{12} \in N(V{\setminus } M_{12})$. We show that $rw(G[M_1 \cup M_2]) \le k$. By assumption, both $G[M_1]$ and $G[M_2]$ have rank-width at most *k*. Since rank-width is preserved by taking induced subgraphs, the graphs $G_{11} = G[M_{11} \cup \{v_{12}\}], G_{12} = G[M_{12} \cup \{v_{22}\}]$, and $G_{22} = G[M_{22} \cup \{v_{12}\}]$ also have rank-width at most *k*. We finish the proof by applying Lemma 4, where $M_{11}, M_{22}, M_{12}$ take the roles of *A*, *B*, and *C* and $v_{12}, v_{12}$, and $v_{22}$ take the roles of *a*, *b*, and *c*, respectively. $\square$

### Definition 7

Let *G* be a graph and $k \in \mathbb {N}$. We define a relation $\sim ^G_k$ on *V*(*G*) by letting $v \sim ^G_k w$ if and only if there is a split-module *M* of *G* such that $v,w \in M$ and $rw(G[M]) \le k$. We drop the superscript from $\sim ^G_k$ if the graph *G* is clear from context.

Using Lemma 6 to deal with transitivity, we prove the following.

### Proposition 2

For every $k \in \mathbb {N}$ and graph $G=(V,E)$ of rank-width at least $k+2$, the relation $\sim _k$ is an equivalence relation, and each equivalence class *U* of $\sim _k$ is a split-module of *G* such that $rw(G[U]) \le k$.

### Proof

Let *G* be a graph and $k \in \mathbb {N}$. For every $v \in V$, the singleton $\{v\}$ is a split-module of *G*, so $\sim _k$ is reflexive. Symmetry of $\sim _k$ is trivial. For transitivity, let $u,v,w \in V$ be such that $u \sim _k v$ and $v \sim _k w$. Then there are split-modules $M_1, M_2$ of *G* such that $u,v \in M_1, v, w \in M_2$, and $rw(G[M_1]), rw(G[M_2]) \le k$; in particular, since $rw(G)\ge k+2$ this implies that there exists a connected component $G'$ of *G* containing *u*, *v*, *w*. By Lemma 6, $M_1 \cup M_2$ is a split-module of $G'$ (and hence also of *G*) such that $rw(G[M_1 \cup M_2]) \le k$. In combination with $u,w \in M_1 \cup M_2$ that implies $u \sim _k w$. This concludes the proof that $\sim _k$ is an equivalence relation.

Now let $v \in V, G'$ be the connected component containing *v*, and let $U = [v]_{\sim _k}$. For each $u \in U$ there is a split-module $W_u$ of $G'$ (and of *G*) such that $u,v \in W_u$ and $rw(G[W_u]) \le k$. By Lemma 6, $W = \bigcup _{u \in U} W_u$ is a split-module of $G'$ (and hence also of *G*) and $rw(G[W]) \le k$. Clearly, $[v]_{\sim _k} \subseteq W$. On the other hand, $u \in W$ implies $v \sim _k u$ by definition of $\sim _k$, so $W \subseteq [v]_{\sim _k}$. That is, $W = [v]_{\sim _k}$. $\square$

### Corollary 2

Every graph *G* of rank-width at least $k+2$ has its vertex set uniquely partitioned by the equivalence classes of $\sim _k$ into inclusion-maximal split-modules of rank-width at most *k*.

Next, we state a simple but useful observation.

### Observation 1

Let $k\in \mathbb {N}, G$ be a disconnected graph having rank-width at least $k+2$, and $\mathcal {C}(G)$ be the set of connected components of *G*. Then $\sim ^G_k=\bigcup _{G' \in \mathcal {C}(G)}\sim ^{G'}_k$.

Now that we know $\sim _k$ is an equivalence relation, we show how to compute it in FPT time. It will be useful to recall Theorems 1 and 2 from Sect. 2.3.

### Proposition 3

Let $k \in \mathbb {N}$. Given an *n*-vertex graph *G* of rank-width at least $k+2$ and two vertices *v*, *w*, we can decide whether $v \sim _k w$ in time $f(k)\cdot n^3$ for some computable function *f*.

### Proof

From Observation 1 it follows that if the proposition holds for connected graphs, then it holds for disconnected graphs as well; hence we may assume that *G* is connected. By Theorem 2 we can compute the unique split-tree $\text {ST}(G)=(T,\mathcal {F})$ in $\mathcal {O}((m+n)\alpha (m+n))$ time. Due to Theorem 1, every split in *G* is the bipartition of leaves of *T* induced either by removing an internal tree-edge of *T* or an edge created by a node-split of a degenerate vertex of *T*.

Vertices of *G* are leaves of *T* and we can find a path *P* between *v* and *w* in *T* in time linear in size of *T*. Since the number of nodes in a split-decomposition is linear in the number of vertices of the original graph [23], there are at most linearly many vertices on the path. Moreover, if $u\in T$ is a degenerate node on *P*, then we can split it into two nodes $u_1, u_2$ in a way such that $G(u_1)$ contains exactly the two marker vertices of *G*(*u*) incident to an edge of *T* on *P* and a new marker vertex connecting it to $u_2$. We split all degenerate nodes on *P* in this way and denote the new tree by $T'$. Note that now every degenerate node on a new path $P'$ between *u* and *v* has 3 vertices.

Now every edge between $P'$ and $T'{\setminus } P'$ corresponds to a minimal split-module containing *v* and *w*. Conversely, as a consequence of Theorem 1 every minimal split-module containing *v* and *w* is induced by removing an edge between $P'$ and $T'{\setminus } P'$, and let $M_{vw}$ be the set containing all of these at most |*T*| minimal split modules. Hence, $v \sim _k w$ if and only if there is a split-module *X* in $M_{vw}$ such that $rw(G[X])\le k$. By Theorem 3 we can decide, for each such *X*, whether $rw(G[X])\le k$ in time $f(k)\cdot n^3$, where *f* is some computable function. $\square$

In the rest of this section we show how to find a *k*-well-structured modulator to any graph class $\mathcal {H}$ characterized by a finite obstruction set $\mathcal {F}$. We first present the algorithm and then show its running time and correctness.
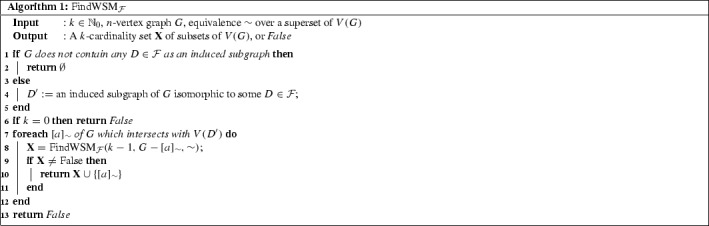


We will use $\sim _k$ as the input for *FindWSM* $_\mathcal {F}$, however considering general equivalence relations as inputs is useful for proving correctness. Recall that the equivalence relation $\sim _k$ (or, more precisely, the set of its equivalence classes) can be computed in time $n^2\cdot f(k)\cdot n^3$ for some function *f* thanks to Proposition 3, and this only needs to be done once before starting the algorithm. The following two lemmas show that Algorithm 1 is correct and runs in FPT time. For fixed $\mathcal {F}$, let $c_\mathcal {F}$ denote the maximum number of vertices of a graph in $\mathcal {F}$.

### Lemma 7

*FindWSM* $_\mathcal {F}$ runs in time $\mathcal {O}(c_\mathcal {F}^k\cdot n^{c_\mathcal {F}})$.

### Proof

The time required to perform the steps on lines $\mathbf {2}$–$\mathbf {6}$ is $\mathcal {O}(n^{c_\mathcal {F}})$ since $\mathcal {F}$ is finite. Similarly, it holds that $|V(D')|$ and hence also the number of times the procedure on lines $\mathbf {8}$–${\mathbf {1}}{\mathbf {3}}$ is called are bounded by $c_\mathcal {F}$.

For the rest of the proof, we proceed by induction on *k*. First, if $k=0$, then the algorithm is polynomial by the above. So assume that $k\ge 1$ and the algorithm for $k-1$ runs in time at most $c_\mathcal {F}^{k-1}\cdot n^{c_\mathcal {F}}$. Then the algorithm for *k* will run in polynomial time up to lines $\mathbf {8}$–$\mathbf {13}$, where it will make at most $c_\mathcal {F}$ calls to the algorithm for $k-1$, which implies that the running time for *k* is bounded by $\mathcal {O}(c_\mathcal {F}^{k}\cdot n^{c_\mathcal {F}})$. $\square$

### Lemma 8

Let $k\ge 0, G$ be a graph and $\sim$ an equivalence relation over a superset of *V*. Then *FindWSM* $_\mathcal {F}(k,G,\sim )$ outputs a set $\mathbf {X}$ of at most *k* equivalence classes of $\sim$ such that $G-\mathbf {X}$ is $\mathcal {F}$-free.

### Proof

If *G* does not contain any *D* as an induced subgraph, then we correctly return the empty set. So, assume there exists an induced subgraph $D'$ of *G* isomorphic to *D*. We prove the lemma by induction on *k*.

Clearly, if $k=0$ but there exists some obstruction, then the algorithm outputs *False* and this is correct; if $k=0$ and no obstruction exists, then the algorithm correctly outputs $\emptyset$. Let $k\ge 1$ and assume that the algorithm is correct for $k-1$. If *G* does not contain any such $\mathbf {X}$, then for any equivalence class $[a]_\sim$, FindWSM$_\mathcal {F}({k-1}, G-[a]_{\sim }, \sim )$ will correctly output *False*.

On the other hand, assume *G* does contain some $\mathbf {X}$ with the desired properties. In particular, this implies that $\mathbf {X}$ must intersect $V(D')$. Let $X_i$ be an arbitrary equivalence class of $\mathbf {X}$ which intersects $V(D')$. Then $\mathbf {X}'{\setminus } \{X_i\}$ is a set of at most $k-1$ equivalence classes of $\sim$ in $G-X_i$, and hence FindWSM$_\mathcal {F}(k-1, G-X'_i, \sim )$ will output some solution $\mathbf {X}''$ for $G-X'_i$ by our inductive assumption. Since any obstruction in *G* intersecting $X'_i$ is removed by $X'_i$ and $G-X'_i$ is made $\mathcal {F}$-free by $\mathbf {X}''$, we observe that $\mathbf {X}''\cup X'_i$ intersects every obstruction in *G* and hence the proof is complete. $\square$

From Lemma 8 and Corollary 2 we obtain the following.

### Corollary 3

Let $k\in \mathbb {N}, G$ be a graph of rank-width at least $k+2$ and $\sim _k$ be the equivalence relation computed by Proposition 3. Then *FindWSM* $_\mathcal {F}(k,G,\sim _k)$ either outputs a *k*-well-structured modulator to $\mathcal {H}$ or correctly detects that no such modulator exists.

### Proof of Theorem 6

Consider the following procedure. First, we test whether *G* has rank-width at most $k+1$ [29], and if this is the case then one can find a *k*-well-structured modulator to $\mathcal {H}$ by using standard techniques. For instance, one may use the extension of Courcelle’s Theorem [11] to *counting monadic second order logic* (CMSO); see for instance the work of Courcelle and Oum [12]. CMSO extends MSO logic by adding atomic formulas which express that the cardinality of a set is divisible by a (fixed) number. The property of “having rank-width at most *k*” is known to be expressible in CMSO logic [12], the properties of “being a split-module” and “not containing any obstruction from a finite set” are easily verified to be expressible in CMSO and even MSO logic, and CMSO logic can be model checked in polynomial time on graphs of bounded rank-width [12, 29].

On the other hand, assume that the input graph *G* has rank-width at least $k+2$. Then the theorem follows by using Proposition 3 and then Algorithm 1 in conjunction with Lemma 7 and Corollary 3. $\square$

## Examples of Algorithmic Applications

This section contains several examples of how the notion of *k*-well-structured modulators can be used to design fixed-parameter algorithms.

### Results for Specific Problems

Our first examples deal with two classical NP-hard graph problems, specifically Minimum Vertex Cover (MinVC) and Maximum Clique (MaxClq). Given a graph *G*, a set $X\subseteq V(G)$ is a *vertex cover* if every edge is incident to at least one $v\in X$ and a *clique* if *G*[*X*] is a complete graph. 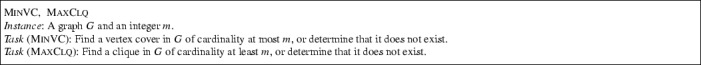


Establishing the following theorem is the main objective of this subsection.

#### Theorem 8

Let $\mathcal {P}\in \{\textsc {MinVC},\textsc {MaxClq}\}$ and $\mathcal {H}$ be a graph class characterized by a finite obstruction set. Then $\mathcal {P}$ is *FPT* parameterized by $wsn^\mathcal {H}$ if and only if $\mathcal {P}$ is polynomial-time tractable on $\mathcal {H}$.

Since $wsn^\mathcal {H}(G)=0$ for any $\mathcal {F}$-free graph *G*, the “only if” direction is immediate; in other words, being polynomial-time tractable on $\mathcal {H}$ is clearly a necessary condition for being fixed-parameter tractable when parameterized by $wsn^\mathcal {H}(G)$. Below we prove that for the selected problems this condition is also sufficient.

#### Lemma 9

If MinVC is polynomial-time tractable on a graph class $\mathcal {H}$ characterized by a finite obstruction set, then $\textsc {MinVC}$ parameterized by $wsn^\mathcal {H}$ is *FPT*.

#### Proof

Let $G=(V,E)$ be a graph and let $k=wsn^\mathcal {H}(G)$. If $rw(G)\le k+2$, then we simply use known algorithms to solve the problem in FPT time [19]. Otherwise, we proceed by using Theorem 6 to compute a *k*-well-structured modulator $\mathbf {X}=\{X_1,\ldots ,X_k\}$ in FPT time. For each $i\in [k]$, we let $A_i$ be the frontier of $X_i$ and we let $B_i=N(A_i)$.

Since for each $i\in [k]$ the graph $G[A_i\cup B_i]$ contains a complete bipartite graph, any vertex cover of *G* must be a superset of either $A_i$ or $B_i$. We can branch over these options for each *i* in $2^k$ time; formally, we branch over all of the at most $2^k$ functions $f:[i]\rightarrow \{A,B\}$, and refer to these as *signatures*. Each vertex cover *Y* of *G* can be associated with at least one signature *f*, constructed in the following way: for each $i\in [k]$ such that $A_i\subseteq Y$, we set $f(i)=A$, and otherwise we set $f(i)=B$.

Our algorithm then proceeds as follows. For a graph *G* and a signature *f*, we construct a partial vertex cover $Z=\bigcup _{i\in [k]} f(i)$. We let $G'=G-Z$. Consider any connected component *C* of $G'$. If *C* intersects some $X_i$, then by the construction of *Z* it must hold that $C\subseteq X_i$. Hence it follows that *C* either has rank-width at most *k* (in the case $C\subseteq X_i$ for some *i*), or *C* is in $\mathcal {H}$ (if *C* does not intersect $\mathbf {X}$), or both. Then we find a minimum vertex cover for each connected component of $G'$ independently, by either calling the known fixed-parameter algorithm (if *C* has bounded rank-width) or the polynomial algorithm (if *C* is in $\mathcal {H}$) at most |*C*| times. Let $Z'$ be the union of the obtained minimum vertex covers over all the components of $G'$, and let $Y_f=Z\cup Z'$. After branching over all possible functions *f*, we compare the obtained cardinalities of $Y_f$ and choose any $Y_f$ of minimum cardinality. Finally, we compare $|Y_f|$ and the value of *m* provided in the input.

We argue correctness in two steps. First, assume for a contradiction that *G* contains an edge *e* which is not covered by $Y_f$ for some *f*. Then *e* cannot have both endpoints in $G'$, since $Y_f$ contains a (minimum) vertex cover for each connected component of $G'$, but *e* cannot have an endpoint outside of $G'$, since $Z\subseteq Y_f$. Hence each $Y_f$ is a vertex cover of *G*.

Second, assume for a contradiction that there exists a vertex cover $Y'$ of *G* which has a lower cardinality than the vertex cover found by the algorithm described above. Let *f* be the signature of $Y'$. Then it follows that $Z\subseteq Y'$, and since $Z\subseteq Y_f$, there would exist a component *C* of $G{\setminus } Z$ such that $|Y'\cap C|\le |Y_f\cap C|$. However, this would contradict the minimality of $Z'\cap C=Y_f\cap C$. Hence we conclude that no such $Y'$ can exist, and the algorithm is correct. $\square$

We deal with the second problem below.

#### Lemma 10

If MaxClq is polynomial-time tractable on a graph class $\mathcal {H}$ characterized by a finite obstruction set, then $\textsc {MaxClq}$ parameterized by $wsn^\mathcal {H}$ is *FPT*.

#### Proof

We begin in the same way as for MinVC: let $G=(V,E)$ be a graph and let $k=wsn^\mathcal {H}(G)$. If $rw(G)\le k+2$, then we simply use known algorithms to solve the problem in FPT time [19]. Otherwise, we proceed by using Theorem 6 to compute a *k*-well-structured modulator $\mathbf {X}=\{X_1,\ldots ,X_k\}$ in FPT time. For each $i\in [k]$, we let $A_i$ be the frontier of $X_i$ and we let $B_i=N(A_i)$.

Let $X_0=G-\mathbf {X}$ and let $s\subseteq \{0\}\cup [k]$. Then any clique *C* in *G* can be uniquely associated with a *signature* *s* by letting $i\in s$ if and only if $X_i\cap C\ne \emptyset$. The algorithm proceeds by branching over all of the at most $2^{k+1}$ possible non-empty signatures *s*. If $|s|=1$, then the algorithm simply computes a maximum-cardinality clique in $X_{s}$ (by calling the respective FPT or polynomial algorithm at most a linear number of times) and stores it as $Y_{s}$.

If $|s|\ge 2$, then the algorithm makes two checks before proceeding. First, if $0\in s$ then it constructs the set $X'_0$ of all vertices $x\in X_0$ such that *x* is adjacent to every $A_i$ for $i\in s{\setminus } \{0\}$. If $X'_0=\emptyset$ then the current choice of *s* is discarded and the algorithm proceeds to the next choice of *s*. Second, for every $a\ne b$ such that $a,b\in s{\setminus } \{0\}$ it checks that $X'_a=A_a$ and $X'_b=A_b$ are adjacent; again, if this is not the case, then we discard this choice of *s* and proceed to the next choice of *s*. Finally, if the current choice of *s* passed both tests then for each $i\in s$ we compute a maximum clique in each $G[X'_i]$ and save their union as $Y_s$. In the end, we choose a maximum-cardinality set $Y_s$ and compare its cardinality to the value of *m* provided in the input.

We again argue correctness in two steps. First, assume for a contradiction that $Y_s$ is not a clique, i.e., there exist distinct non-adjacent $a,b\in Y_s$. Since $Y_s$ consists of a union of cliques within subsets of $X'_{i\in s}$, it follows that there would have to exist distinct $c,d\in s$ such that $a\in X'_c$ and $b\in X'_d$. This can however be ruled out for *c* or *d* equal to 0 by the construction of $X'_0$. Similarly, if *c* and *d* are both non-zero, then this is impossible by the second check which tests adjacency of every pair of $X'_c$ and $X'_d$ for every $c,d\in s$.

Second, assume for a contradiction that there exists a clique $Y'$ in *G* which has a higher cardinality than the largest clique obtained by the above algorithm. Let *s* be the signature of $Y'$. If $|s|=1$ then $|Y_s|\ge |Y'|$ by the correctness of the respective FPT or polynomial algorithm used for each $X_s$. If $|s|\ge 2$ then $Y'$ may only intersect the sets $X'$ constructed above for *s*. Moreover, if there exists $i\in [k]\cup \{0\}$ such that $|Y'\cap X'_i|> |Y_s\cap X'_i|$ then we again arrive at a contradiction with the correctness of the respective FPT or polynomial algorithms used for $X'_i$. Hence we conclude that no such $Y'$ can exist, and the algorithm is correct. $\square$

**Fig. 4:**
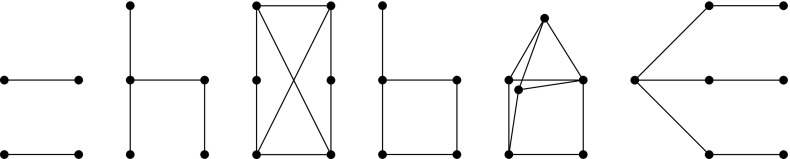
From *left to right* $2K_2$, fork, $K_{3,3}\text {-}e$, banner, twin-house, and $T_{2,2,2}$

Finally, let us review some concrete graph classes for use in Theorem 8. We use $K_i, C_i$ and $P_i$ to denote the *i*-vertex complete graph, cycle, and path, respectively. $2K_2$ denotes the disjoint union of two $K_2$ graphs. The *fork*, $K_{3,3}\text {-}e$, *banner*, *twin-house* and $T_{2,2,2}$ graphs are depicted in Fig. 4.

#### Fact 2

MinVC is polynomial-time tractable on the following graph classes:

1. $(2K_2,C_4,C_5)$-free graphs (split graphs);
2. $$P_5$$-free graphs;
3. fork-free graphs;
4. $(\text {banner}, T_{2,2,2})$-free graphs and $(\text {banner}, K_{3,3}\text {-}e, \text {twin-house})$-free graphs.

#### Proof

For item 1, recall that split graphs are graphs whose vertex set can be partitioned into one clique and one independent set, and such a partitioning can be found in linear time. If each vertex in the clique is adjacent to at least one independent vertex, then the clique is a minimum vertex cover, otherwise the clique without a pendant-free vertex is a minimum vertex cover. Item 2 follows from [33]. Item 3 follows from [1]. Item 4 follows from [24] and [5]. $\square$

#### Fact 3

MaxClq is polynomial-time tractable on the following graph classes:

1. Any complementary graph class to the classes listed in Fact 2 (such as cofork-free graphs and split graphs);
2. Graphs of bounded degree.

#### Proof

1. It is well-known that each maximum clique corresponds to a maximum independent set (and vice-versa) in the complement graph.
2. The degree bounds the size of a maximum clique, again resulting in a simple folklore branching algorithm. The class of graphs of degree at most *d* is exactly the class of $\mathcal {F}$-free graphs for $\mathcal {F}$ containing all $(d+1)$-vertex supergraphs of the star having *d* leaves. $\square$

### Results for Other Graph Classes

Next, we turn our attention to computing *k*-well-structured modulators to examples of graph classes which are not characterized by a finite obstruction set (i.e., by a finite set of forbidden induced subgraphs). In the following lemmas, *n* denotes the size of the vertex set of the input graph.

#### Lemma 11

It is possible to compute a *k*-well-structured modulator to the class of forests in time $f(k)\cdot n^5$ for some computable function *f*.

#### Proof

We begin by describing our algorithm ${\mathcal {A}}$, and then proceed to argue correctness and runtime bounds. ${\mathcal {A}}$ begins by checking whether the rank-width of the input graph *G* is at least $k+2$; if not, then a *k*-well-structured modulator can be computed using Courcelle’s Theorem in time at most $f(k)\cdot n^3$ for some computable function *f*. ${\mathcal {A}}$ then proceeds in four steps.

- First, it uses Proposition 3 to partition *V*(*G*) into equivalence classes of $\sim _k$ in time at most $f(k)\cdot n^5$ for some computable function *f*, and sets $j:=k; S:=\emptyset ; \sim :=\sim _k$.
- Second, for each tuple (*X*, *Y*, *Z*) of equivalence classes of $\sim , {\mathcal {A}}$ checks whether $G[X\cup Y\cup Z]$ is acyclic. If this is not the case, then ${\mathcal {A}}$ chooses (by branching) one class out of $\{X,Y,Z\}$ to delete from $\sim$, saves the deleted equivalence class in *S*, and restarts the second step with $j:=j-1$. If $j=-1$, then the algorithm terminates the given branch.
- Third, ${\mathcal {A}}$ constructs an auxiliary graph $G'=(V',E')$ by setting $V'$ to be the set of equivalence classes of $\sim$ and $E'$ to contain an edge between $A,B\in V'$ iff there exist vertices $a\in A, b\in B$ such that $ab\in E(G)$.
- Finally, ${\mathcal {A}}$ tries to find a feedback vertex set in $G'$ of size at most *j* in time $\mathcal {O}(3.83^j\cdot j|V'|^{2})$ [7]. If no such feedback vertex set exists, then ${\mathcal {A}}$ terminates the given branch; otherwise it adds the feedback vertex set to $\mathbf {S}$ and outputs $\mathbf {S}$.

It is easy to verify that the running time of ${\mathcal {A}}$ is upper-bounded by $\mathcal {O}(f(k)\cdot n^5)$ for some computable function *f*. As for correctness, let us assume for a contradiction that ${\mathcal {A}}$ outputs a set $\mathbf {S}$ and the graph *H* obtained from *G* after deleting all vertices in elements of $\mathbf {S}$ contains a cycle *C*. Clearly, neither *C* nor any other cycle in *H* intersects less than 4 equivalence classes of $\sim _k$, since otherwise such a cycle would have been detected and removed in step 2 of ${\mathcal {A}}$.

Moreover, assume $|C\cap X|>1$ for some equivalence class *X* of $\sim _k$. Since *C* spans at least 4 equivalence classes, *H* must contain at least two neighbors of *X* in $C{\setminus } X$ which are adjacent to at least two vertices in *X* (indeed, recall that *X* is a split-module and hence all vertices of *X* with a neighbor outside *X* have the same neighborhood outside *X*); let us denote these vertices $y,z,x_1,x_2$, respectively. Since $x_1, x_2$ are in the frontier of *X*, the vertices $y,x_1,z,x_2$ must form a cycle in *H* which spans at most 3 equivalence classes, contradicting our previous conclusion that no such cycles are present in *H*. Hence we may conclude that $|C\cap X|\le 1$ for every equivalence class *X*.

The only case we are left with now is that *C* intersects each equivalence class at most once. But then it must be the case that *C* also forms a cycle in $G'$, which would have necessarily been removed in step 4 of ${\mathcal {A}}$, a contradiction. So *H* must indeed be acyclic.

For the other direction, assume that *G* contains a minimal *k*-well-structured modulator $\mathbf {X}=\{X_1,\ldots , X_j\}$ to the class of forests. Then consider the branch of step 2 of ${\mathcal {A}}$ which hits a maximal number of elements of $\mathbf {X}$, and let us denote the elements removed by ${\mathcal {A}}$ in this way $\mathbf {Y}$. By the same argument as above, each cycle remaining in *G* after deleting $\mathbf {Y}$ intersects each equivalence class at most once and hence corresponds to a cycle in the graph $G'$ constructed by ${\mathcal {A}}$. In particular, the equivalence classes in $\mathbf {X}{\setminus } \mathbf {Y}$ form a feedback vertex set of size $\ell =|\mathbf {X}{\setminus } \mathbf {Y}|$ in $G'$. By the correctness of the feedback vertex set algorithm used in step 4, at least one branch of ${\mathcal {A}}$ is guaranteed to output a solution $\mathbf {S}\supset \mathbf {Y}$ of size at most *j*. $\square$

For the next result, recall that a cycle is *chordless* if it is also an induced cycle of length at least 4, and a graph is *chordal* if it contains no chordless cycles.

#### Lemma 12

It is possible to compute a *k*-well-structured modulator to the class of chordal graphs in time $f(k)\cdot n^{\mathcal {O}(1)}$.

#### Proof

We once again first describe our algorithm ${\mathcal {A}}$. ${\mathcal {A}}$ begins by checking whether the rank-width of the input graph *G* is at least $k+2$; if not, then a *k*-well-structured modulator can be computed using Courcelle’s Theorem in time at most $f(k)\cdot n^3$ for some computable function *f*. ${\mathcal {A}}$ then proceeds in four steps.

- First, it uses Proposition 3 to partition *V*(*G*) into equivalence classes of $\sim _k$ in time at most $f(k)\cdot n^5$, and sets $j:=k; S:=\emptyset ; \sim :=\sim _k$.
- Second, for each tuple (*X*, *Y*, *Z*) of equivalence classes of $\sim , {\mathcal {A}}$ checks whether $G[X\cup Y\cup Z]$ is chordal in linear time [36]. If this is not the case, then ${\mathcal {A}}$ chooses (by branching) one class out of $\{X,Y,Z\}$ to delete from $\sim$, saves the deleted equivalence class in *S*, and restarts the second step with $j:=j-1$. If $j=-1$, then the algorithm terminates the given branch.
- Third, ${\mathcal {A}}$ constructs an auxiliary graph $G'=(V',E')$ by setting $V'$ to be the set of equivalence classes of $\sim$ and $E'$ to contain an edge between $A,B\in V'$ iff there exist vertices $a\in A, b\in B$ such that $ab\in E(G)$.
- Finally, ${\mathcal {A}}$ tries to find a modulator to chordal graphs of size at most *j* in $G'$ using the algorithm by Marx [8], which takes time at most $2^{\mathcal {O}(k\log k)}\cdot |V'|^{\mathcal {O}(1)}$. If no such modulator exists, then ${\mathcal {A}}$ terminates the given branch; otherwise it adds the modulator to $\mathbf {S}$ and outputs $\mathbf {S}$.

It is easy to verify that the running time of ${\mathcal {A}}$ is upper-bounded by $f(k)\cdot n^{\mathcal {O}(1)}$ for some computable function *f*. As for correctness, let us assume for a contradiction that ${\mathcal {A}}$ outputs a set $\mathbf {S}$ and the graph *H* obtained from *G* after deleting all vertices in elements of $\mathbf {S}$ contains a chordless cycle *C*; without loss of generality, let us assume *C* is such a chordless cycle of minimum length. Clearly, neither *C* nor any other chordless cycle in *H* intersects less than 4 equivalence classes of $\sim _k$, since otherwise such a cycle would have been detected and removed in step 2 of ${\mathcal {A}}$.

We now claim that *C* contains at most one vertex from each equivalence class of $\sim _k$. To see this, assume for a contradiction that *C* contains two vertices in some equivalence class *Z*. Since *C* must also intersect other equivalence classes, it follows that *C* must in fact contain at least two vertices, say *x*, *y*, in the frontier of *Z* which have distinct neighbors, say $x', y'$ respectively, in $C{\setminus } Z$. First, observe that *x*, *y* cannot occur consecutively along *C*, as that would violate the assumption that *C* is a minimum-length chordless cycle. Hence by the chordality of *C* we also see that *xy* cannot be an edge of *G*, and the same also applies for the non-edge of $x'y'$. But then $x',x,y',y$ forms a chordless cycle which intersects at most 3 equivalence classes, contradicting our previous assumptions.

Let us now consider the set $C'$ of equivalence classes which intersect *C*; recall that $|C'|>3$. By the above claim, it follows that $C'$ would also be a chordless cycle in $V(G')$, contradicting the correctness of the chordal vertex deletion algorithm [8]. Hence we conclude that *H* must in fact be chordal.

For the other direction, assume that *G* contains a minimal *k*-well-structured modulator $\mathbf {X}=\{X_1,\ldots , X_j\}$ to the class of chordal graphs. Then consider the branch of step 2 of ${\mathcal {A}}$ which hits a maximal number of elements of $\mathbf {X}$, and let us denote the elements removed by ${\mathcal {A}}$ in this way $\mathbf {Y}$. By the same argument as above, each chordless cycle remaining in *G* after deleting $\mathbf {Y}$ intersects each equivalence class at most once and hence corresponds to a chordless cycle in the graph $G'$ constructed by ${\mathcal {A}}$. In particular, the equivalence classes in $\mathbf {X}{\setminus } \mathbf {Y}$ form a modulator to chordal graphs of size $\ell =|\mathbf {X}{\setminus } \mathbf {Y}|$ in $G'$. By the correctness of the chordal vertex deletion algorithm used in step 4, at least one branch of ${\mathcal {A}}$ is guaranteed to output a solution $\mathbf {S}\supset \mathbf {Y}$ of size at most *j*. $\square$

## MSO Model Checking with Well-Structured Modulators

Here we show how well-structured modulators can be used to solve the MSO model checking problem, as formalized in Theorem 9 below. Note that our meta-theorem captures not only the generality of MSO model checking problems, but also applies to a potentially unbounded number of choices of the graph class $\mathcal {H}$. Thus, the meta-theorem supports two dimensions of generality.

### Theorem 9

Let $\phi$ be a ${\text {MSO}}$ sentence and $\mathcal {H}$ be a graph class characterized by a finite obstruction set. If $\textsc {MSO-MC}_{\phi }$ is *FPT* parameterized by $mod^\mathcal {H}(G)$, then $\textsc {MSO-MC}_{\phi }$ is also *FPT* parameterized by $wsn^\mathcal {H}(G)$.

The condition that $\textsc {MSO-MC}_{\phi }$ is FPT parameterized by $mod^\mathcal {H}(G)$ is a necessary condition for the theorem to hold by Proposition 1. However, it is natural to ask whether it is possible to use a weaker necessary condition instead, specifically that $\textsc {MSO-MC}_{\phi }$ is polynomial-time tractable on $\mathcal {H}$ (as was done for specific problems in Sect. 5). Before proceeding towards a proof of Theorem 9, we make a digression and show that the weaker condition used in Theorem 8 is in fact not sufficient for the general case of MSO model checking.

### Lemma 13

There exists an MSO sentence $\phi$ and a graph class $\mathcal {H}$ characterized by a finite obstruction set such that $\textsc {MSO-MC}_{\phi }$ is polynomial-time tractable on $\mathcal {H}$ but NP-hard on the class of graphs having $wsn^\mathcal {H}(G)\le 2$ and even $mod^\mathcal {H}(G)\le 2$.

### Proof

Consider the sentence $\phi$ which describes the existence of a proper 5-coloring of the vertices of *G*, and let $\mathcal {H}$ be the class of graphs of degree at most 4 (in other words, let $\mathcal {F}$ contain all 6-vertex supergraphs of the star having 5 leaves). There exists a trivial greedy algorithm to obtain a proper 5-coloring of any graph of degree at most 4, hence $\textsc {MSO-MC}_{\phi }$ is polynomial-time tractable on $\mathcal {H}$. Now consider the class of graphs obtained from $\mathcal {H}$ by adding, to any graph in $\mathcal {H}$, two adjacent vertices *y*, *z* which are both adjacent to every other vertex in the graph. By construction, any graph $G'$ from this new class satisfies $mod^\mathcal {H}(G')\le 2$ and hence also $wsn^\mathcal {H}(G')\le 2$. However, $G'$ admits a proper 5-coloring if and only if $G'-\{y,z\}$ admits a proper 3-coloring. Testing 3-colorability on graphs of degree at most 4 is known to be NP-hard [30], and hence the proof is complete. $\square$

Our strategy for proving Theorem 9 relies on a replacement technique, where each split-module in the well-structured modulator is replaced by a small representative. We use the notion of *similarity* defined below to prove that this procedure does not change the outcome of $\textsc {MSO-MC}_{\varphi }$.

### Definition 8

*(Similarity)* Let *q* and *k* be non-negative integers, $\mathcal {H}$ be a graph class, and let *G* and $G'$ be graphs having *k*-well-structured modulators $\mathbf {X}=\{X_1,\ldots ,X_k\}$ and $\mathbf {X'}=\{X_1',\ldots ,X_k'\}$ to $\mathcal {H}$, respectively. For $1\le i\le k$, let $S_i$ contain the frontier of split module $X_i$ and similarly let $S'_i$ contain the frontier of split module $X'_i$. We say that $(G, \mathbf {X})$ and $(G', \mathbf {X}')$ are *q*-*similar* if all of the following conditions are met:

1. There exists an isomorphism $\tau$ between $G-\mathbf {X}$ and $G'-\mathbf {X}'$.
2. For every $v\in V(G){\setminus } \mathbf {X}$ and $i\in [k]$, it holds that *v* is adjacent to $S_i$ if and only if $\tau (v)$ is adjacent to $S'_i$.
3. If $k\ge 2$, then for every $1\le i<j\le k$ it holds that every $s_i\in S_i$ is adjacent to every $s_j\in S_j$ if and only if every $s'_i\in S'_i$ is adjacent to every $s'_j\in S'_j$.
4. For each $$i\in [k]$$, it holds that $$ type _q(G[X_i],S_i)= type _q(G'[X'_i],S'_i)$$.

### Lemma 14

Let *q* and *k* be non-negative integers, $\mathcal {H}$ be a graph class, and let *G* and $G'$ be graphs having *k*-well-structured modulators $\mathbf {X}=\{X_1,\ldots ,X_k\}$ and $\mathbf {X'}=\{X_1',\ldots ,X_k'\}$ to $\mathcal {H}$, respectively. If $(G, \mathbf {X})$ and $(G', \mathbf {X}')$ are *q*-*similar*, then it holds that $type _q(G, \emptyset ) = type _q(G', \emptyset )$.

### Proof

For $i \in [k]$, we write $G_i = G[X_i]$ and $G_i' = G'[X_i']$. Let $X_0=V(G){\setminus } \mathbf {X}$ and $X'_0=V(G'){\setminus } \mathbf {X'}$. By Theorem 5, Condition 4 of Definition 8 is equivalent to $(G_i, S_i) \equiv ^{{\text {MSO}}}_q (G'_i, S'_i)$. That is, for each $i \in [k]$, Duplicator has a winning strategy $\pi _i$ in the *q*-round MSO game played on $G_i$ and $G_i'$ starting from $(S_i, S'_i)$. We construct a strategy witnessing $(G, \emptyset ) \equiv ^{{\text {MSO}}}_q (G', \emptyset )$ in the following way:

1. Suppose Spoiler makes a set move *W* and assume without loss of generality that $W \subseteq V(G)$. For $i \in [k]$, let $W_i = X_i \cap W$, and let $W_i'$ be Duplicator’s response to $W_i$ according to $\pi _i$. Furthermore, let $W'_0=\{\,\tau (v)\;{|}\;v\in W\cap X_0 \,\}$. Then Duplicator responds with $W' = W'_0 \cup \bigcup _{i=1}^k W_i'$.
2. Suppose Spoiler makes a point move *s* and again assume without loss of generality that $s \in V(G)$. If $s \in X_i$ for some $i \in [k]$, then Duplicator responds with $s' \in X_i'$ according to $\pi _i$; otherwise, Duplicator responds with $\tau (s)$ as per Definition 8 item 1.

Assume Duplicator plays according to this strategy and consider a play of the *q*-round MSO game on *G* and $G'$ starting from $(\emptyset , \emptyset )$. Let $\mathbf {v}=(v_1,\ldots ,v_m)$ and $\mathbf {u}=(u_1,\ldots ,u_m)$ be the point moves in *V*(*G*) and $V(G')$ respectively, and let $\mathbf {V}=(V_1, \ldots , V_l)$ and $\mathbf {U}=(U_1,\ldots , U_l)$ be the set moves in *V*(*G*) and $V(G')$ respectively, so that $l + m = q$ and the moves made in the same round have the same index. We claim that $(\mathbf {v},\mathbf {u})$ defines a partial isomorphism between $(G,\mathbf {V})$ and $(G',\mathbf {U})$.

- Let $j_1,j_2 \in [m]$ and let $v_{j_1}, v_{j_2}\in X_0$. Since $\tau$ is an isomorphism as per Definition 8 item 1, it follows that $v_{j_1}=v_{j_2}$ if and only if $u_{j_1} = u_{j_2}$ and $v_{j_1}v_{j_2}\in E(G)$ if and only if $u_{j_1}u_{j_2}\in E(G')$.
- Let $j_1,j_2 \in [m]$ and let $i\in [k]$ be such that $v_{j_1}\in X_0$ and $v_{j_2}\in X_i$. Then clearly $v_{j_1}\ne v_{j_2}$ and $u_{j_1}\ne u_{j_2}$. Consider the case $v_{j_1}v_{j_2}\in E(G)$. Then $v_{j_2}$ must lie in the frontier of $X_i$, and hence $v_{j_2}\in S_i$. Since Duplicator’s strategy $\pi _i$ is winning for $(G_i,S_i)$ and $(G'_i,S'_i)$, it must hold that $u_{j_2}\in S'_i$. By Definition 8 item 2, it then follows that $\tau (v_{j_1})u_{j_2}\in E(G')$. So, consider the case $v_{j_1}v_{j_2}\not \in E(G)$. Then either $v_{j_2}\not \in S_i$, in which case it holds that $u_{j_2}\not \in S'_i$ because of the choice of $\pi _i$ and hence there cannot be an edge $u_{j_2}u_{j_1}$ in $G'$, or $v_{j_2}\in S_i$, in which case it holds once again that $u_{j_2}u_{j_1}\not \in E(G')$ by Definition 8 item 2.
- Let $j_1,j_2 \in [m]$ and let $i \in [k]$ be such that $v_{j_1},v_{j_2} \in X_{i}$. Since Duplicator plays according to a winning strategy $\pi _i$ in the game on $G_i$ and $G_i'$, the restriction $(\mathbf {v}|_i, \mathbf {u}|_i)$ defines a partial isomorphism between $(G_i, (\mathbf {V})|_i)$ and $(G_i', (\mathbf {U})|_i)$. It follows that $(v_{j_1},v_{j_2}) \in E(G)$ if and only if $(u_{j_1},u_{j_2}) \in E(G')$ and $v_{j_1} = v_{j_2}$ if and only if $u_{j_1} = u_{j_2}$.
- Let $j_1,j_2 \in [m]$ and let $i_1,i_2 \in [k]$ be pairwise distinct numbers such that $v_{j_1}\in X_{i_1}$ and $v_{j_2}\in X_{i_2}$. Then $v_{j_1} \ne v_{j_2}$ and also $u_{j_1} \ne u_{j_2}$ since $u_{j_1} \in X_{i_1}'$ and $u_{j_2} \in X_{i_2}'$ by the Duplicator’s strategy. Suppose $v_{j_1}v_{j_2}\in E(G)$. Then $v_{j_1}\in S_{i_1}$, and $v_{j_2}\in S_{i_2}$, and $S_{i_1}$ and $S_{i_2}$ are adjacent in *G*. From the correctness of $\pi _{i_1}$ and $\pi _{i_2}$ it follows that $u_{j_1}\in S'_{i_1}$ and $u_{j_2}\in S'_{i_2}$, and from Definition 8 item 3 it follows that $S'_{i_1}$ and $S'_{i_2}$ are adjacent in $G'$, which together implies $u_{j_1}u_{j_2}\in E(G')$. On the other hand, suppose $v_{j_1}v_{j_2}\not \in E(G)$. Then either $v_{j_1}\not \in S_{i_1}$, or $v_{j_2}\not \in S_{i_2}$, or $S_{i_1}$ and $S_{i_2}$ are not adjacent in *G*. In the first case we have $u_{j_1}\not \in S'_{i_1}$, in the second case we have $u_{j_2}\not \in S'_{i_2}$, and in the third case it holds that $S'_1$ and $S'_2$ are not adjacent in $G'$; any of these three cases imply $u_{j_1}u_{j_2}\not \in E(G')$.
- Let $j \in [m]$ such that $v_j\in X_0$. Then by the Duplicator’s strategy on $X_0$ it follows that for any $V_q$ such that $v_j\in V_q$ it holds that $u_j\in U_q$ and for any $V_q$ such that $v_j\not \in V_q$ it holds that $u_j\not \in U_q$.
- Let $j\in [m]$ and $i\in [k]$ such that $v_j\in X_k$. Let $V_q$ be such that $v_j\in V_q$. Since $\pi _i$ is a winning strategy for Duplicator, it must be the case that $u_j\in U_q$. Similarly, if $v_j\not \in V_q$ then the correctness of $\pi _i$ guarantetes that $u_j\not \in U_q$. $\square$

Next, we show that small representatives can be computed efficiently.

### Lemma 15

Let $$q\in \mathbb {N}_0$$. There exist functions *f*, *g* such that one can compute, for an input graph *G* of rank-width at most *k* and $$S\subseteq V(G)$$, in time $$f(k)\cdot |V(G)|^{\mathcal {O}(1)}$$ a graph $$G'$$ and a set $$S'\subseteq V(G')$$ such that $$|V(G')|\le g(q)$$ and $$ type _q(G,S) = type _q(G',S')$$.

### Proof

By Lemma 1 we can compute a formula $\varPhi (Q)$ capturing the type *T* of (*G*, *S*) in time $f(k)\cdot |V(G)|^{\mathcal {O}(1)}$. Given $\varPhi (Q)$, a constant-size model $(G',S')$ satisfying $\varPhi (Q)$ can be computed as follows. We start enumerating all graphs (by brute force and in any order with a non-decreasing number of vertices), and check for each graph $G^*$ and every vertex-subset $S^*\subseteq V(G^*)$ whether $G^* \models \varPhi (S^*)$. If this is the case, we stop and output $(G^*,S^*)$. Since $G \models \varPhi (S)$ this procedure must terminate eventually. Fixing the order in which graphs are enumerated, the number of graphs we have to check depends only on *T*. By Fact 1 the number of *q*-types is finite for each *q*, so we can think of the total number of checks and the size of each checked graph $G^*$ as bounded by a constant. Moreover the time spent on each check depends only on *T* and the size of the graph $G^*$. Consequently, after we compute $\varPhi (Q)$ it is possible to find a model for $\varPhi (Q)$ in constant time. $\square$

Finally, in Lemma 16 below we use Lemma 15 to replace any well-structured modulator by a small but “equivalent” modulator.

### Lemma 16

Let *q* be a non-negative integer constant and $\mathcal {H}$ be a graph class. Then given a graph *G* and a *k*-well-structured modulator $\mathbf {X}=\{X_1,\ldots X_k\}$ of *G* into $\mathcal {H}$, there exists a function *f* such that one can in time $f(k)\cdot |V(G)|^{\mathcal {O}(1)}$ compute a graph $G'$ with a *k*-well-structured modulator $\mathbf {X'}=\{X'_1,\ldots X'_k\}$ into $\mathcal {H}$ such that $(G,\mathbf {X})$ and $(G',\mathbf {X'})$ are *q*-similar and for each $i \in [k]$ it holds that $|X_i'|$ is bounded by a constant.

### Proof

For $i\in [k]$, let $S_i\subseteq X_i$ be the frontier of split-module $X_i$, let $G_i=G[X_i]$ and let $G_0=G{\setminus } G[\mathbf {X}]$. We compute a graph $G_i'$ of constant size and a set $S_i'\subseteq V(G_i')$ having the same MSO *q*-type as $(G_i,S_i)$. By Lemma 15, this can be done in time $f(k)\cdot |V(G)|^{\mathcal {O}(1)}$ for some function *f*. Now let $G'$ be the graph obtained by the following procedure:

1. We construct a disjoint union of $G_0$ and $G'_i$ for each $i\in [k]$;
2. If $k\ge 2$ then for each $1\le i<j \le k$ such that $S_i$ and $S_k$ are adjacent in *G*, we add edges between every $v\in S'_i$ and $w\in S'_j$.
3. for every $v\in V(G_0)$ and $i\in [k]$ such that $S_i$ and $\{v\}$ are adjacent, we add edges between *v* and every $w\in S'_i$.

It is easy to verify that $(G,\mathbf {X})$ and $(G', \mathbf {X}')$, where $\mathbf {X}'=\{V(G_1'),\ldots ,V(G_k')\}$, are *q*-similar. $\square$

### Proof of Theorem 9

Let *G* be a graph, $k=wsn^\mathcal {H}(G)$ and *q* be the nesting depth of quantifiers in $\phi$. By Theorem 6 it is possible to find a *k*-well-structured modulator to $\mathcal {H}$ in time $f(k)\cdot |V|^{\mathcal {O}(1)}$. We proceed by constructing $(G',\mathbf {X}')$ by Lemma 16. Since each $X'_i\in \mathbf {X}'$ has size bounded by a constant and $|\mathbf {X}'|\le k$, it follows that $\bigcup \mathbf {X}'$ is a modulator to the class of $\mathcal {F}$-free graphs of cardinality $\mathcal {O}(k)$. Hence $\textsc {MSO-MC}_{\phi }$ can be decided in FPT time on $G'$. Finally, since *G* and $G'$ are *q*-similar, it follows from Lemma 14 that $G\models \phi$ if and only if $G'\models \phi$. $\square$

For completeness, we remark that the same proof can be used to obtain analogues of Theorem 9 for any graph class $\mathcal {H}$ which admits a fixed-parameter algorithm for finding well-structured modulators (i.e., even if it is not characterized by a finite obstruction set; see for instance Lemma 11 and 12).

We conclude the section by showcasing an example application of Theorem 9. *c*-Coloring asks whether the vertices of an input graph *G* can be colored by *c* colors so that each pair of neighbors have distinct colors. From the connection between *c*-Coloring, its generalization List *c* -Coloring and modulators [6, Theorem 3.3] and tractability results for List *c* -Coloring [28, Page 5], we obtain the following.

### Corollary 4

For each $c\in \mathbb {N}, c$-Coloring parameterized by $wsn^{P_5\text {-free}}$ is *FPT*.

## Hardness of MSO Optimization

In the wake of Theorem 9 and the positive results for the two problems in Sect. 5, one would expect that it should be possible to strengthen Theorem 9 to also cover LinEMSO problems [11, 19], which extend MSO model checking by allowing the minimization/maximization of linear expressions over free set variables. Surprisingly this is not possible if we wish to retain the same necessary conditions, as will be shown in this section. For our proof, it suffices to consider a simplified variant of LinEMSO, defined below. Let $\varphi$ be an MSO formula with one free set variable.

$$\textsc {MSO-Opt}^{\le }_{\varphi }$$

*Instance*: A graph *G* and an integer $r\in \mathbb {N}$.

*Question*: Is there a set $S \subseteq V(G)$ such that $G \models \varphi (S)$ and $|S| \le r$?

We say that $S\subseteq V(G)$ is a *dominating set* if every vertex in *G* either is in *S* or has a neighbor in *S*. We will need the following lemma before we proceed to the main result of this secton.

### Lemma 17

The problem of finding a *p*-cardinality dominating set in a graph *G* having a *k*-cardinality modulator $X\subseteq V(G)$ to the class of graphs of degree at most 3 is FPT when parameterized by $p+k$.

### Proof

Let $L=V(G){\setminus } X$ and consider the following algorithm. We begin with $D=\emptyset$, and choose an arbitrary vertex $v\in L$ which is not yet dominated by *D*. We branch over the at most $k+4$ vertices *q* in $\{v\}\cup N(v)$, and add *q* to *D*. If $|D|=p$ and there still exists an undominated vertex in *G*, we discard the current branch; hence this procedure produces a total of at most $(k+4)^p$ branches.

Now consider a branch where $|D|<p$ but the only vertices left to dominate lie in *X*. For $a,b\in L$, we let $a\equiv b$ if and only if $N(a)\cap X=N(b)\cap X$. Notice that $\equiv$ has at most $2^k$ equivalence classes and that these may be computed in polynomial time. For each non-empty equivalence class of $\equiv$, we choose an arbitrary representative and construct the set *P* of all such chosen representatives. We then branch over all subsets *Q* of $P\cup X$ of cardinality at most $p-|D|$, and add *Q* into *D*. Since $|P\cup X|\le 2^k+k$, this can be done in time bounded by $\mathcal {O}(2^{p\cdot k})$. Finally, we test whether this *D* is a dominating set, and output the minimum dominating set obtained in this manner.

It is easily observed from the description that the running time is FPT. For correctness, from the final check it follows that any set outputed by the algorithm will be a dominating set. It remains to show that if there exists a dominating set of cardinality *p*, then the algorithm will find such a set. So, assume there exists a *p*-cardinality dominating set $D'$ in *G*. Consider the branch arising from the first branching rule obtained as follows. Let $v_1$ be the first undominated vertex in *L* chosen by the algorithm, and consider the branch where an arbitrary $q\in D'\cap N(v_1)$ is placed into *D*. Hence, after the first branching, there is a branch where $D\subseteq D'$. Similarly, there exists a branch where $D\subseteq D'$ for each $v_i$ chosen in the *i*-th step of the first branching. If $D'=D$ after the first branching, then we are done; so, let $D'_1=D'{\setminus } D$ be non-empty. Let $D_1$ be obtained from $D'_1$ by replacing each $w\in D'_1$ by the representative of $[w]_\equiv$ chosen to lie in *P*. Since $D'$ dominates all vertices in *L* and $D_1$ dominates the same vertices in *X* as $D'_1$, it follows that $D^*=(D'{\setminus } D'_1)\cup D_1$ is also a dominating set of *G*. Furthermore, $|D^*|=|D'|$. However, since $D_1\subseteq P$ and $|D_1|\le p-|D|$, there must exist a branch in the second branching which sets $Q=D_1$. Hence there exists a branch in the algorithm which obtains and outputs the set $D^*=D\cup D_1$. $\square$

### Theorem 10

There exists an MSO formula $\varphi$ and a graph class $\mathcal {H}$ characterized by a finite obstruction set such that $\textsc {MSO-Opt}^{\le }_{\varphi }$ is *FPT* parameterized by $mod^\mathcal {H}$ but *paraNP*-hard parameterized by $wsn^\mathcal {H}$.

### Proof

To prove Theorem 10, we let *dom*(*S*) express that *S* is a dominating set in *G*, and let *cyc*(*S*) express that *S* intersects every $C_4$ (cycle of length 4). Then we set $\varphi (S)=dom(S)\vee cyc(S)$ and let $\mathcal {H}$ be the class of $C_4$-free graphs of degree at most 3 (obtained by letting the obstrucion set $\mathcal {F}$ contain $C_4$ and all 5-vertex supergraphs of $K_{1,4}$).

### Claim

$\textsc {MSO-Opt}^{\le }_{\varphi }$ is FPT parameterized by the cardinality of a modulator to $\mathcal {H}$.

To argue that the above claim holds, let $(G=(V,E),r)$ be the input of $\textsc {MSO-Opt}^{\le }_{\varphi }$ and *k* be the cardinality of a modulator in *G* to $\mathcal {H}$. We begin by computing some modulator $X\subseteq V$ of cardinality *k* in *G* to $\mathcal {H}$; this can be done in FPT time by a simple branching algorithm on any of the obstructions from $\mathcal {F}$ located in *G*. Let $L=V{\setminus } X$. Next, we compare *r* and *k*, and if $r\ge k$ then we output YES. This is correct, since each $C_4$ in *G* must intersect *X* and hence setting $S=X$ satisfies $\varphi (S)$.

So, assume $r<k$. Then we check whether there exists a set *A* of cardinality at most *r* which intersects every $C_4$; this can be done in time $O^*(4^r)$ by a simple FPT branching algorithm. Next, we check whether there exists a dominating set *B* in *G* of cardinality at most *r*; this can also be done in FPT time by Lemma 17.

Finally, if *A* or *B* exists, then we output YES and otherwise we output NO. Hence the claim is indeed true.

### Claim

$\textsc {MSO-Opt}^{\le }_{\varphi }$ is paraNP-hard parameterized by $wsn^\mathcal {H}(G)$.

We proceed by arguing that this claim is also correct. It is known that the Dominating Set problem, which takes as input a graph *G* and an integer *j* and asks to find a dominating set of size at most *j*, is NP-hard on $C_4$-free graphs of degree at most 3 [31] (see also subsequent work [2, Theorem 8]). We use this fact as the basis of our reduction. Let (*G*, *j*) be a $C_4$-free instance of Dominating Set of degree at most 3. Then we construct $G'$ from *G* by adding $(|G|+2)$-many copies of $C_4$, a single vertex *q* adjacent to every vertex of every such $C_4$, and a single vertex $q'$ adjacent to *q* and an arbitrary vertex of *G*. It is easy to check that $wsn^\mathcal {H}(G')\le 2$.

We claim that (*G*, *j*) is a YES-instance of Dominating Set if and only if $(G',j+1)$ is a yes-instance of $\textsc {MSO-Opt}^{\le }_{\varphi }$. For the forward direction, assume there exists a dominating set *D* in *G* of cardinality *j*. Then the set $D\cup \{q\}$ is a dominating set in $G'$, and hence satisfies $\varphi$.

On the other hand, assume there exists a set $D'$ of cardinality at most $j+1$ which satisfies $\varphi$. If $j+1\ge |G|+2$ then clearly (*G*, *j*) is a YES-instance of Dominating Set, so assume this is not the case. But then $D'$ cannot intersect every $C_4$, and hence $D'$ must be a dominating set of $G'$ of cardinality at most $j+1$. But this is only possible if $q\in D'$. Furthermore, if $q'\in D'$, then replacing $q'$ with the neighbor of $q'$ in *G* is also a dominating set of $G'$. Hence we may assume, w.l.o.g., that $D'\cap V(G)$ is a dominating set of cardinality at most *j* in *V*(*G*). Consequently, (*G*, *j*) is a YES-instance of Dominating Set and the claim holds.

The theorem now follows from the two claims proved above.$\square$

## Conclusion

We have introduced a family of structural parameters which push the frontiers of fixed-parameter tractability beyond rank-width and modulator size for a wide range of problems. In particular, the well-structure number can be computed efficiently (Theorem 6) and used to design fixed-parameter algorithms for Minimum Vertex Cover and Maximum Clique (Theorem 8) as well as any problem which can be described by a sentence in MSO logic (Theorem 9). We remark that while our results are of a theoretical nature, there is hope that some of the ideas behind the presented algorithms may be useful in practice once faster algorithms for computing rank-width become available.

For future work, it would be interesting to see whether the notion of split-modules introduced in this work can be naturally generalized. In particular, a split-module *X* can be seen as a subgraph such that $\varvec{A}_G[X,G-X]=1$, and in this sense split-decompositions naturally correspond to rank-width 1. It is easy to define corresponding decompositions also for higher values of rank-width, however it is not at all clear how such decompositions could be computed. We believe this is an interesting question on its own; furthermore, obtaining such decompositions would allow an immediate extension of our framework to the arising more general notions of split-modules.

Finally, we remark that well-structured modulators have also found applications in the area of data reduction and *kernelization* [16]. In particular, since $wsn^{\mathcal {H}}$ lower-bounds rank-width and rank-width is known not to admit polynomial kernels for nearly any NP-hard problems, one cannot hope to use $wsn^{\mathcal {H}}$ for polynomial kernelization. However, a number of polynomial kernels have been developed for a restriction of $wsn^{\mathcal {H}}$ where each split module induces a graph whose rank-width is bounded by a constant [17].
